# Small Molecule-Mediated Stage-Specific Reprogramming of MSCs to Hepatocyte-Like Cells and Hepatic Tissue for Liver Injury Treatment

**DOI:** 10.1007/s12015-024-10771-x

**Published:** 2024-09-11

**Authors:** Santosh Gupta, Akriti Sharma, Muthukumarassamy Rajakannu, Jovana Bisevac, Mohamed Rela, Rama Shanker Verma

**Affiliations:** 1 Stem Cell and Molecular Biology, Laboratory, Department of Biotechnology, Bhupat and Jyoti Mehta School of Biosciences, Indian Institute of Technology Madras, Chennai, Tamil Nadu 600036 India; 2 Centre for Eye Research and Innovative Diagnostics, Department of Ophthalmology, Institute of Clinical Medicine, University of Oslo, Oslo, Norway; 3 The Institute of Liver Disease & Transplantation, Dr. Rela Institute & Medical Centre, Bharath Institute of Higher Education & Research, Chromepet, Tamil Nadu India

**Keywords:** Hepatocyte-like cells (dHep), Small molecules, Mesenchymal stem cells, Decellularization, Hepatic tissue

## Abstract

**Background:**

Derivation of hepatocytes from stem cells has been established through various protocols involving growth factor (GF) and small molecule (SM) agents, among others. However, mesenchymal stem cell-based derivation of hepatocytes still remains expensive due to the use of a cocktail of growth factors, and a long duration of differentiation is needed, thus limiting its potential clinical application.

**Methods:**

In this study, we developed a chemically defined differentiation strategy that is exclusively based on SM and takes 14 days, while the GF-based protocol requires 23–28 days.

**Results:**

We optimized a stage-specific differentiation protocol for the differentiation of rat bone marrow-derived mesenchymal stem cells (MSCs) into functional hepatocyte-like cells (dHeps) that involved four stages, i.e., definitive endoderm (DE), hepatic competence (HC), hepatic specification (HS) and hepatic differentiation and growth. We further generated hepatic tissue using human decellularized liver extracellular matrix and compared it with hepatic tissue derived from the growth factor-based protocol at the transcriptional level. dHep, upon transplantation in a rat model of acute liver injury (ALI), was capable of ameliorating liver injury in rats and improving liver function and tissue damage compared to those in the ALI model.

**Conclusions:**

In summary, this is the first study in which hepatocytes and hepatic tissue were derived from MSCs utilizing a stage-specific strategy by exclusively using SM as a differentiation factor.

**Graphical Abstract:**

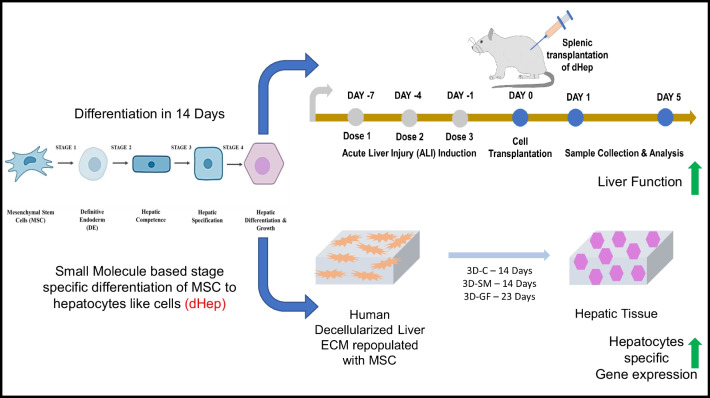

**Supplementary Information:**

The online version contains supplementary material available at 10.1007/s12015-024-10771-x.

## Introduction

Hepatocytes, the major parenchymal cells in terms of function and volume in the liver, are essential for regenerative treatment of liver-related acute and chronic diseases as a cell therapy module [1, 2]. The development of hepatocyte-like cells has been explored using a variety of stem cells [3], such as induced pluripotent stem cells (iPSCs) [4], embryonic stem cells (ESCs) [5] and mesenchymal stem cells (MSCs) [6, 7]. These stem cell-derived hepatocytes, like cells, are unlimited sources of hepatocytes that are useful for potential clinical applications. However, such cell types are associated with limitations such as genomic instability, high production costs, variable gene expression, scale-up limitations (in the case of iPSCs and ESCs) [8], loss of stemness and long culture times (in the case of MSCs) [9]. Another limitation is the use of a growth factor-based differentiation protocol that increases the cost of hepatocyte derivation. Recently, several studies have emphasized the use of small molecules (SMs) for deriving functional hepatocytes, such as cells from iPSCs [10], ESCs [11] and MSCs [12]. These works mimic the induction/regulation of various phases of liver development in utero to generate functional hepatocytes, similar to cells [13]. Interestingly, MSCs are a large source of adult stem cells with extensive evidence of hepatogenesis under defined conditions of differentiation. However, compared with iPSCs, MSCs have not been explored extensively owing to limitations such as extended culture time, costly differentiation protocols and extended differentiation duration (approximately 28 days) for the derivation of hepatocyte-like cells [6, 14, 15]. iPSCs and ESCs have been used for hepatocyte-like cell derivation via the use of a cocktail of SM with direct or stage-specific differentiation strategies [16]. However, such protocols have not been explored for MSCs, which can be used in clinical settings owing to their genomic stability and nonteratogenic behavior, which are exhibited by iPSCs and ESCs.

Strategies such as direct and stage-specific differentiation of iPSCs have been used to derive hepatocytes using small molecules (SM) to eventually reduce the differentiation time and cost of deriving hepatocytes such as cells at a large scale [16]. SM are chemical low molecular weight organic molecules that exhibits pharmacological effect by its inhibitory or excitatory effect by binding to specific protein targets either on the cell surface receptors or intracellular targets to manifest cellular effects [13]. However, the relevance of iPSCs in the clinic for liver regeneration is still debatable, and iPSCs are currently underexplored as the most promising option [17]. Recently, SM has been used for deriving hepatocyte-like cells from MSCs. Itaba et al. showed that a single SM can be used to generate hepatocyte-like cells. However, such derived cells exhibit hepatocyte functionality [12]. However, these cells have not been tested for long-term inductive effects, which is one of the concerns related to single-molecule-based directed differentiation. A stage-specific differentiation protocol is desirable because it modulates the gene expression profile related to each stage of liver development. In such cases, the derived differentiated cells might resemble the primary hepatocyte transcriptome and proteome more closely [18].

Once a protocol is optimized, it can be used for the development of hepatic tissue using biomaterial-based scaffolds [19]. There are a variety of natural and synthetic biomaterial-based scaffolds used for the development of hepatic tissue as well as whole liver organs that exhibit hepatic function [20]. In this regard, decellularized liver organ-derived extracellular matrix is an ideal candidate for the development of hepatic tissue because the architecture and biochemical composition of the ECM resemble or are identical to those of the native liver extracellular milieu [21]. Several studies have shown the advantage of using decellularized liver ECM as a scaffold for hepatic tissue development. A study by Jiang et al. showed that, compared with 2D differentiation, differentiation of MSCs in mouse decellularized liver tissue resulted in more efficient hepatic tissue, and upon transplantation, it improved the survival of mice with liver injury [22]. Therefore, for potential clinical application, the use of human liver-derived extracellular matrix can potentially be used for hepatic tissue development because it is allogenic and can tolerate the immune response [23, 24].

To the best of our knowledge, this is the first study to show the derivation of functional hepatocyte-like cells (dHeps) from MSCs in a stage-specific manner using SM. The stage-specific gene expression and protein marker expression of the derived dHeps were studied using real-time PCR, immunocytochemistry, and flow cytometry. dHeps were characterized for their functional SM-based derivation compared with GF-based stage-specific derivation at the gene and protein levels. We further used human liver-derived extracellular matrix (ECM) and developed hepatic tissue using our optimized protocol and compared it with growth factor-derived hepatic tissue. To assess the therapeutic potential of dHep, we performed splenic transplantation of dHep in an acute liver injury (ALI) model in rats and studied the therapeutic ability of dHep to ameliorate liver injury and promote liver tissue regeneration. Taken together, these findings showed that SM-mediated stage-specific differentiation of MSCs into dHeps can be used to ameliorate acute liver injury in a rat model. Our protocol can be used to develop hepatic tissue samples that can potentially be used in clinical application.

## Materials and Methods

### Materials

Dulbecco’s modified Eagle’s medium Ham F 12 (DMEM-F12) was purchased from Invitrogen Life Technologies (Gibco; Carlsbad, CA, USA). Fetal bovine serum (FBS), L-glutamine, penicillin/streptomycin antibiotics (AB), trypsin/EDTA and phosphate-buffered saline (PBS) were purchased from Gibco (USA). Fluorescein isothiocyanate (FITC)-conjugated anti-mouse/rat, rat mesenchymal stem cell osteogenic, chondrogenic and adipogenic differentiation media were purchased from Gibco, USA. All the other chemicals were obtained from standard laboratory suppliers and were of the highest purity available. The present study was approved by the ethics committee of the Indian Institute of Technology Madras (India).

### Methods

#### Animal and Human Ethics Approval

Technical and ethical clearance data for the use of animals were obtained from the Institute Animal Ethical Committee (IAEC) of the Indian Institute of Technology (IIT) Madras, India, following established conventions and regulations (Animal study: Approval no—IEC/2018/01/RSV-6/10). All the experiments were performed in accordance with the Helsinki Declaration. All the procedures in the experiment were performed according to the protocol approved by the ethical committee of IIT Madras, India.

#### Isolation and Characterization of Rat Bone Marrow MSCs (MSCs)

Rat bone marrow MSCs were isolated from Wistar rats (4 weeks). The detailed procedure used for isolation, culture, expansion and characterization has been described in the supplementary information datasheet. The isolated MSC were characterized for CD 105, CD 90 and CD 45 (SI Fig. 1) according to the ISCT guidelines.

#### Differentiation of MSCs in 2D Culture Conditions

**Fig. 1:**
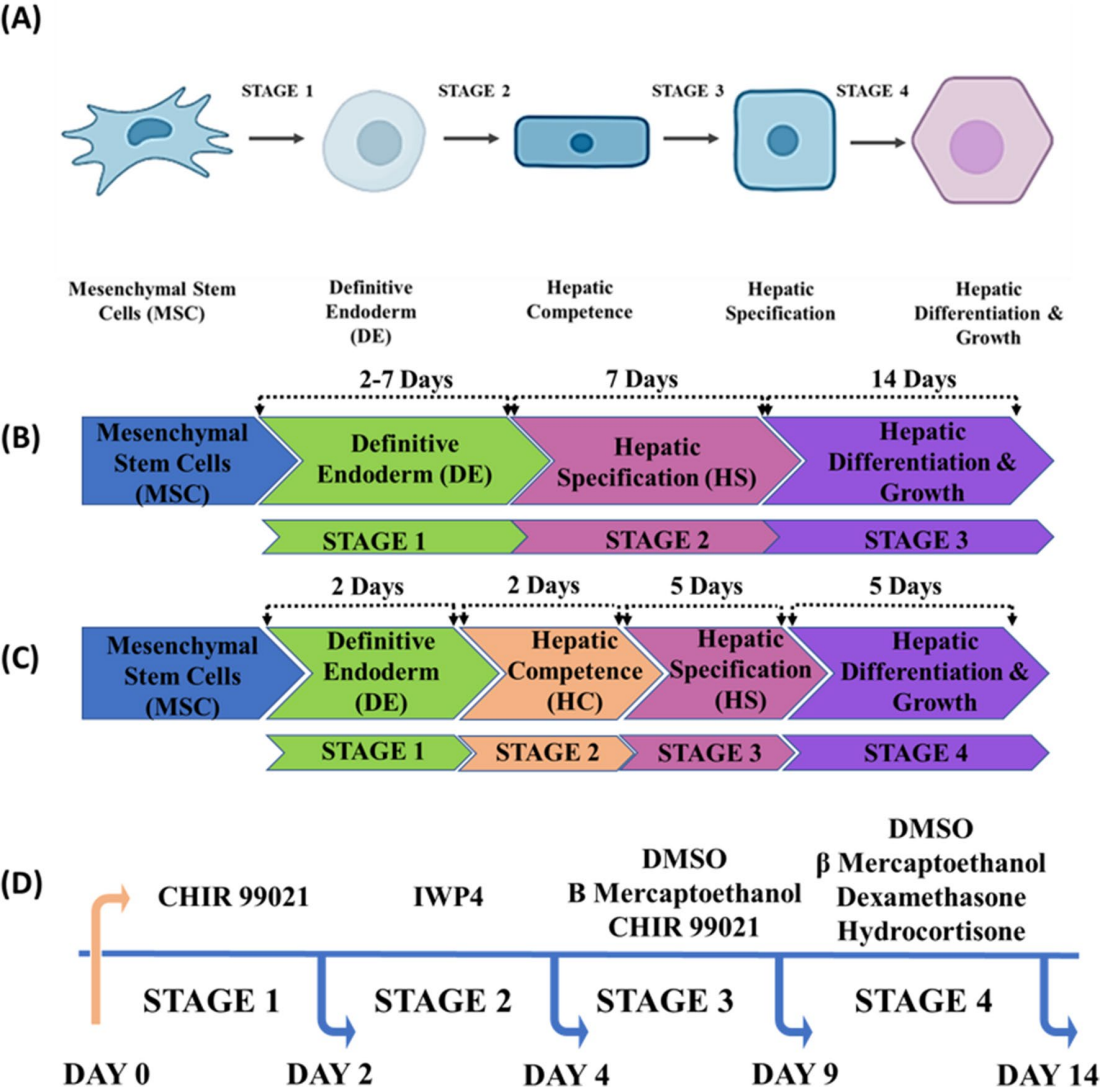
**A** Schematic representation of the stage-specific differentiation of hepatocytes developed in this study. Chronological representation of the stages employed in the differentiation of hepatocytes using (**B)** established growth factor-based protocol and (**C**). small molecules (SM) in the present study Description of the use of SM in the present study along with the optimized time taken to induce stage-specific cell types for hepatocyte deviation (**D**)

MSCs from passages 2–6 were used for all the differentiation studies. MSCs were seeded in a gelatin (0.5%) coated 6-well plate. A total of 1 × 10^6^ cells were seeded in each well and maintained for 2 days. After 48 h, the cells were serum starved in serum-free DMEM-F12 media supplemented with all the supplements for 24 h (day 0). The differentiation protocol started on day 1 according to the schematics presented above. After each differentiation stage, the cells were harvested for immunocytochemistry and qRT‒PCR studies. All differentiation media were freshly prepared. For stage 3 and 4, DMSO and βME were added to the respective media as per the composition prior to media change (Fig. 1).

##### STAGE 1 – Definitive Endoderm (DE)

Methodology- MSCs were used from passages P2 to P6 for the differentiation studies. Briefly, 30,000 cells/well were seeded in a 12-well plate. After 24 h, the cells were subjected to serum-free conditions for 48 h. After serum-free media conditioning, the cells were differentiated on day 1. CHIR 99021 was used at two different concentrations (5 µM and 10 µM), and the induction of definite endoderm was studied for 5 days by real-time PCR. The media was changed after 48 h once during the 5-day study period. After every 24 h, the cells were subjected to total RNA extraction and cDNA conversion. Gene expression analysis specific to the definitive endoderm was performed to assess DE induction based on the highest gene expression of all the genes studied in both the 5 µM and 10 µM groups. Non-induced/nontreated BM-MSCs cultured in 2% FBS were used as controls. All the experiments were performed in triplicate for statistical significance. After analysis of the RT‒PCR results via a heatmap of the expression of all the genes, the treatment day was optimized, and immunocytochemistry and flow cytometry were subsequently performed to assess the protein markers present and to quantify the expression of those proteins, respectively.

##### STAGE 2 – Hepatic Competence (HC)

Methodology- For stage 2 differentiation, 30,000 cells/well were seeded in a 12-well plate. After 24 h, the cells were subjected to serum-free conditions for 48 h. After 48 h, following the optimized no. of days for stage 1, the second stage was induced using IWP4 at two different concentrations (5 µM and 10 µM). The cells were cultured via the same process, which was applied for the stage 1 differentiation strategy. After every 24 h, the cells were subjected to total RNA extraction and cDNA conversion. cDNA real-time studies were performed to determine the gene expression of stage 2-specific genes. Similarly, after optimizing the number of days with the highest expression of stage 2-related genes, the concentration of IWP4 and the number of days were finalized for downstream differentiation. Protein markers and quantitative estimates of stage 2-specific proteins were obtained by immunocytochemistry and flow cytometry, respectively, to confirm the presence of stage 2-related proteins, and quantitative information on stage 2 differentiation efficiency was calculated. All the experiments were performed in triplicate.

##### STAGE 3- Hepatic Specification

Methodology- To induce hepatic specification, cells were treated with dimethyl sulfoxide (DMSO), beta-mercaptoethanol (βME) and CHIR 99021 (2 µM) for 5 days. For stage 3 induction, instead of optimizing the number of days, we kept the number of days fixed at 5 days as shown in previous studies [10, 25, 26]. This condition has been shown to efficiently indue hepatic specified cells. After 5 days of treatment with DMSO, βME or CHIR 99021, total RNA was isolated using TRIzol reagent, and 2 µg of RNA was converted to cDNA. Furthermore, cDNA was used for real-time studies to evaluate the expression of stage 3-related genes. To further quantify the proteins and determine their differentiation efficiency into stage 3 cells, a flow cytometry study was performed. Immunocytochemistry was performed to assess the presence of stage 3 protein markers. All the experiments were performed in triplicate.

##### STAGE 4—Hepatic Differentiation and Growth

Methodology- To induce hepatic differentiation, maturation and growth, stage 4 differentiation media was composed of hydrocortisone, dexamethasone (Dex), dimethyl sulfoxide (DMSO), insulin-transferrin-selenium (ITS), and knockout serum (KOSR); the detailed media composition is provided in the supplementary information file. Briefly, 30,000 cells/well were seeded in 12-well plates, and the stage-specific differentiation strategy was performed according to the optimized conditions for a total of 3 days. After inducing stage 3, i.e., after day 9, stage 4 was induced using the abovementioned stage 4 induction chemical media. After 5 days of differentiation, the cells were subjected to total RNA extraction, and 2 µg of total RNA was used for cDNA conversion. Real-time PCR was performed to assess the relative gene expression patterns of stage 4-related genes. Similarly, upon optimization of stage 4 induction by RT‒PCR, protein markers and quantitative estimates of stage 4-specific proteins were obtained by immunocytochemistry and flow cytometry, respectively, to confirm the presence of stage 4-related proteins, and quantitative information related to stage 4 differentiation efficiency was calculated.

#### Decellularization of the Human Liver

Patients who underwent liver transplantation were used for liver retrieval in the experiments. The portal vein was cannulated with a silicon catheter (4 mm OD), and the cannulated liver was perfused with Hank’s balanced slat solution (HBSS) with heparin (1000 units). The cannulated human liver lobe was subjected to washing with sterilized DPBSA for 2 h using a peristaltic pump operating at a 50 ml/minute flow rate. After washing with DPBSA, the liver was decellularized using a detergent-based method. The details of the decellularization and sterilization procedures are described in the supplementary material. Briefly, 1% SDS was used for 2 h, followed by incubation in Triton X-100 for 2 h. After complete decellularization, the liver was washed with DPBSA for 2 h. After perfusion with DPBSA, the whole liver was sterilized using sterilization buffer containing 0.015% PAA and 4% ethanol. After complete decellularization, the biochemical composition and complete decellularization of the decellularized liver extracellular matrix were characterized.

##### Characterization of Decellularized Liver Extracellular Matrix (DLEM)

For characterization of complete decellularization and its biochemical composition, DLEMs were subjected to various tests. The detailed experimental procedures used for all the experiments are described in the supplementary material. For biochemical analysis, total DNA content was estimated using a genomic DNA isolation kit (Qiagen, USA). For total GAG estimation of the decellularized liver ECM, total GAGs were isolated using the protocol described previously, with minor modifications [27]. Histological analysis was performed to assess the completeness of the cell removal. The microstructure of the DL-ECM was assessed by scanning electron microscopy (SEM).

#### In vitro3D Hepatic Differentiation of MSCs

MSCs were subjected to hepatic transdifferentiation using SM and GF. DLEM (1 × 1x0.5 mm) was placed in a 24-well plate and conditioned in supplemented alpha MEM for 24 h. A total of 5 × 10^5^ MSCs were seeded per well on the DLEM for differentiation studies. After 24 h of culture, the cells were cultured in serum-free media supplemented with a-MEM for 24 h. After serum-free media treatment, the cells were cultured according to an optimized protocol for SM in the present study, and a growth factor-based protocol was established for hepatic transdifferentiation [7, 28, 29].

#### Semiquantitative Real-Time PCR

Semiquantitative real-time PCR (RT–PCR) was performed with the corresponding primers for the specific cell types listed in (SI-Tables 2 and 6) using a Dynamo Color Plus SYBR Green Real-time PCR Kit (Thermo Fisher Scientific, USA). A two-step cycling protocol was performed consisting of a single 10 min cycle at 95 °C, followed by 40 cycles of 10 s at 95 °C and 30 s at 60 °C. Melting curve analysis was performed after amplification to check for the presence of any spurious amplification or the formation of primer dimers. Relative mRNA expression was determined by normalization against the expression of the housekeeping gene GAPDH, and the fold change in expression was subsequently calculated via the 2-ΔΔCt method.

#### Immunofluorescence

Native MSCs and SM- and GF-derived dHep cells were fixed with 4% paraformaldehyde solution for 20 min at room temperature and permeabilized with 0.25% Triton X-100 in 1 × PBS solution for 20 min. The fixed cells were washed with DPBS three times and incubated with blocking buffer containing 5% normal goat serum for 1 h at room temperature. Furthermore, the cells were incubated with primary antibody overnight at 4 °C with gentle shaking (for each stage, see SI Table 3), and the dilutions used are listed in SI Table 7. Then, the cells were washed with DPBS and incubated again for the next 2 h with the appropriate secondary antibody (IgG Alexa Fluor 594/IgG-FITC at a 1:1000 dilution) at room temperature. Nuclei were stained with Hoechst 33,258 dye (Sigma Aldrich, Merck, USA). The stained cells were observed under a fluorescence microscope (Nikon Ti-E, Japan) at 10 × magnification.

#### Flow Cytometry Analysis

After the completion of differentiation for the SM- and GF-based protocols, the cells were harvested by trypsin digestion, and approximately 4 × 105 cells were fixed with 4% paraformaldehyde solution for 20 min and washed twice with DPBSA. For 20 min, the fixed cells were permeabilized with 0.25% Triton X-100. Subsequently, the cells were blocked with 5% normal goat serum for 60 min before being stained with primary monoclonal antibodies (SI Tables 4, 7) for 16 h at 40 °C. Following treatment, the cells were washed in 1 × DPBS and stained with secondary antibodies conjugated to Alexa Fluor 595 and Alexa Fluor 488 (Invitrogen) along with Hoechst 33,342 (Sigma Aldrich, Merck, USA) for 2 h at room temperature (RT) in the dark. Flow cytometry was used for immunophenotyping (BD FACS CantoTM II, BD Biosciences, USA), and the results were analyzed using Flow Jo v.10.0.8 software.

#### Functional Study

##### Albumin and Urea Estimation

The spent media from the control, SM- and GF-derived dHeps were collected after 24 h and used for albumin quantification using a mouse ALB ELISA kit (Cat. No. ab108791; Abcam, UK) according to the manufacturer’s protocol. Untreated cells were used as a negative control for the assay. For urea concentration estimation, the spent media from MSCs (untreated or induced to differentiate) was collected after 24 h and used for the estimation of urea concentration via a urea assay kit (Cat. no. ab83362; Abcam, UK) according to the manufacturer’s protocol. Untreated cells were used as a negative control for the assay.

#### Rat Acute Liver Injury (ALI) Model

To induce acute liver injury in the rat model, CCl4 was used at a concentration of 2 ml/kg of body weight. A total of 40 male rats (10 control and 30 ALI model) were used for the study. CCl_4_ and olive oil were mixed at a ratio of 1:1 and injected into the rats intraperitoneally 3 times at intervals of 2 days. A total of 3 doses of CCl_4_ were injected. The serum was used for liver function tests to assess the extent of injury caused by the CCl_4_ injection. Rats were caged in a institute approved animal house. Rats were fed animal diet and ad libidum.

#### Transplantation of Hepatocyte-Like Cells (dHeps) and MSC in an ALI Model

Twenty-four hours after the 3rd dose of CCL_4_, 5 × 10^6^ dHep and MSC were injected into the spleens of the ALI rats. The cells were counted and suspended in α-MEM without FBS. Briefly, rats were anesthetized by ketamine and xylazine. The left lateral region was shaved and sterilized using povidone solution. A longitudinal incision was made, and the spleen was located. The spleen was retrieved using blunt end forceps, and 5 × 10^6^ dHep and MSC resuspended in 50 µl of α-MEM were injected into the spleen with a 29G needle. In sham control, 50 µl of α-MEM was injected into the spleen. The spleen was checked for retrograde release of the cells by dabbing the area with a moistened cotton swab. The peritoneum was closed, and the skin was sutured with a 4–0 absorbable suture. The sutured skin was sterilized with povidone solution and dressed. The rats were administered antibiotics (1 ml of a 12 mg/ml solution of cefotaxime) intraperitoneally and 1 mg/kg body weight meloxicam for postoperative care.

### Statistical Analysis

All the data are presented as the mean ± SD. All the in vitro experiments were performed in triplicate for statistical validation, and reproducibility was assessed by repeating each experiment at least three times. D’Agostino–Pearson test for normality was performed before statistical analysis among various groups using one-way or two-way analysis of variance (ANOVA) followed by Tukey’s post hoc test using GraphPad Prism software (version 9, USA). Total GAG concentrations were quantified using the Mann‒Whitney U test. Differences were considered to be statistically significant at *p < 0.05, **p < 0.1, and ***p < 0.001. The data are presented as the means of three independent experiments, as mentioned in each figure panel. For heatmap analysis, HeatMapper software was used [30]. Clustering was average linked, and the Pearson method was used for distance measurement.

## Results

### Definitive Endoderm (DE) Induction by a Wnt Activator – Stage 1

**Fig. 2:**
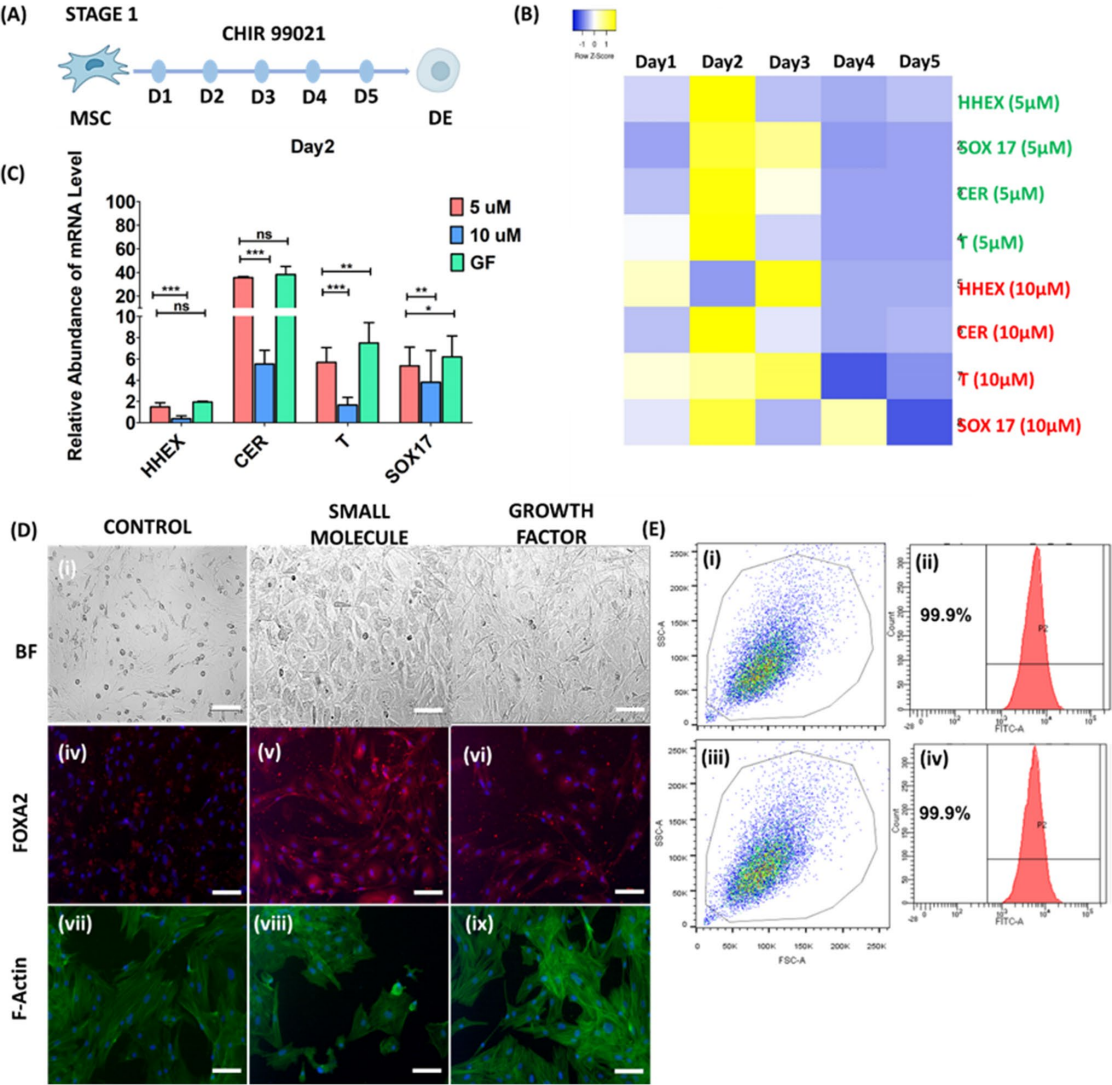
Definitive endoderm (DE) induction by CHIR 99021 – Stage 1. **A** Schematic representation of the definitive endoderm (DE) induction of MSCs. **B** Heatmap analysis of DE-specific gene expression (HHEX, CER, T and SOX17) in the CHIR-99021-treated MSCs. MSCs were treated with CHIR-99021 at 5 µM and 10 µM for 5 days, and real-time PCR was performed to assess the modulation of gene expression. **C** DE-specific gene expression analysis on day 2 in the 5 µM, 10 µM CHIR-99021 and growth factor-based stage 1 protocols (EGF and bFGF) showing that the 5 µM CHIR-99021 and GF protocols had comparable DE-specific gene expression profiles. **D** Immunocytochemistry of DE cells on day 2 after CHIR-99021 treatment compared with that after the GF-based stage 1 protocol. Bright field image of control MSCs (i), DE cells derived from cells treated with CHIR 99021 (ii), and DE cells derived from GF-treated cells (iii). Immunostaining for FOXA2 in MSCs (iv), DE cells derived from cells treated with CHIR 99021 (v), and DE cells derived from GF-treated cells (vi). Actin filament staining by phalloidin-FITC for assessing morphological changes in MSCs (vii), DE cells derived from cells treated with CHIR 99021 (viii), and DE cells derived from GF-treated cells (ix). **E** DE-specific expression analysis of FoxA2 by flow cytometry for the definitive endoderm derived from the CHIR 99021 (i) and GF (ii) cohorts. The data were analyzed by one-way ANOVA. Differences were considered to be statistically significant at *p < .01, **p < .001, and ***p < .0001. The data are presented as the means of three independent experiments; the error bars represent the SDs. Scale bar—100 µm

In vertebrae, the liver arises from the definitive endoderm, which is characterized by the expression of markers such as HHEX, CER, T and SOX17. In our differentiation strategy, to derive the definitive endoderm, CHIR 99021 was used at two different concentrations, 5 µM and 10 µM, to optimize its concentration for inducing the DE stage (Fig. 2A). Gene expression was assessed for DE marker induction every 24 h for 5 days (SI Fig. 2). A heatmap analysis revealed that, out of the two concentrations, 5 µM had higher expression of HHEX, CER, T and SOX17 on day 2 (*p* < 0.001) than 10 µM had no effect on DE-specific gene expression (Fig. 2B). DE induction using the GF protocol did not significantly differ for HHEX or CER gene expression compared to that in the 5 µM CHIR 99021 group on day 2 (Fig. 2C). T and SOX17 gene expression was significantly greater (*p* < 0.01) in the GF group than in the 5 µM CHIR 99021 group (Fig. 2B). Immunocytochemical studies of DE markers revealed positive staining for FOXA2 proteins in SM- and GF-induced DE on day 2 of the induction procedure (Fig. 2D (v)-SM and (vi)-GF). The proteins that were transcription factors showed nuclear localization, indicating the activity of both TFs. The morphology of the MSCs started to change, as observed by actin filament staining (Fig. 2D (viii)-SM and (ix)-GF). The change in fibroblastic morphology indicated changes in the phenotype of the MSCs that underwent DE commitment, signifying the induction of cellular changes toward epithelial morphology, as exhibited during the developmental stages when DE matures from the endodermal germ layer. The expression efficiency of FOXA2, as assessed by flow cytometry, was 99.9 ± 1% and 99.1 ± 1% for SM and GF, respectively (Fig. 2E). FOXA2 expression did not significantly differ between the SM- and GF-based differentiation approaches.

### Hepatic Commitment by the Wnt Inhibitor IWP4: Stage 2

**Fig. 3:**
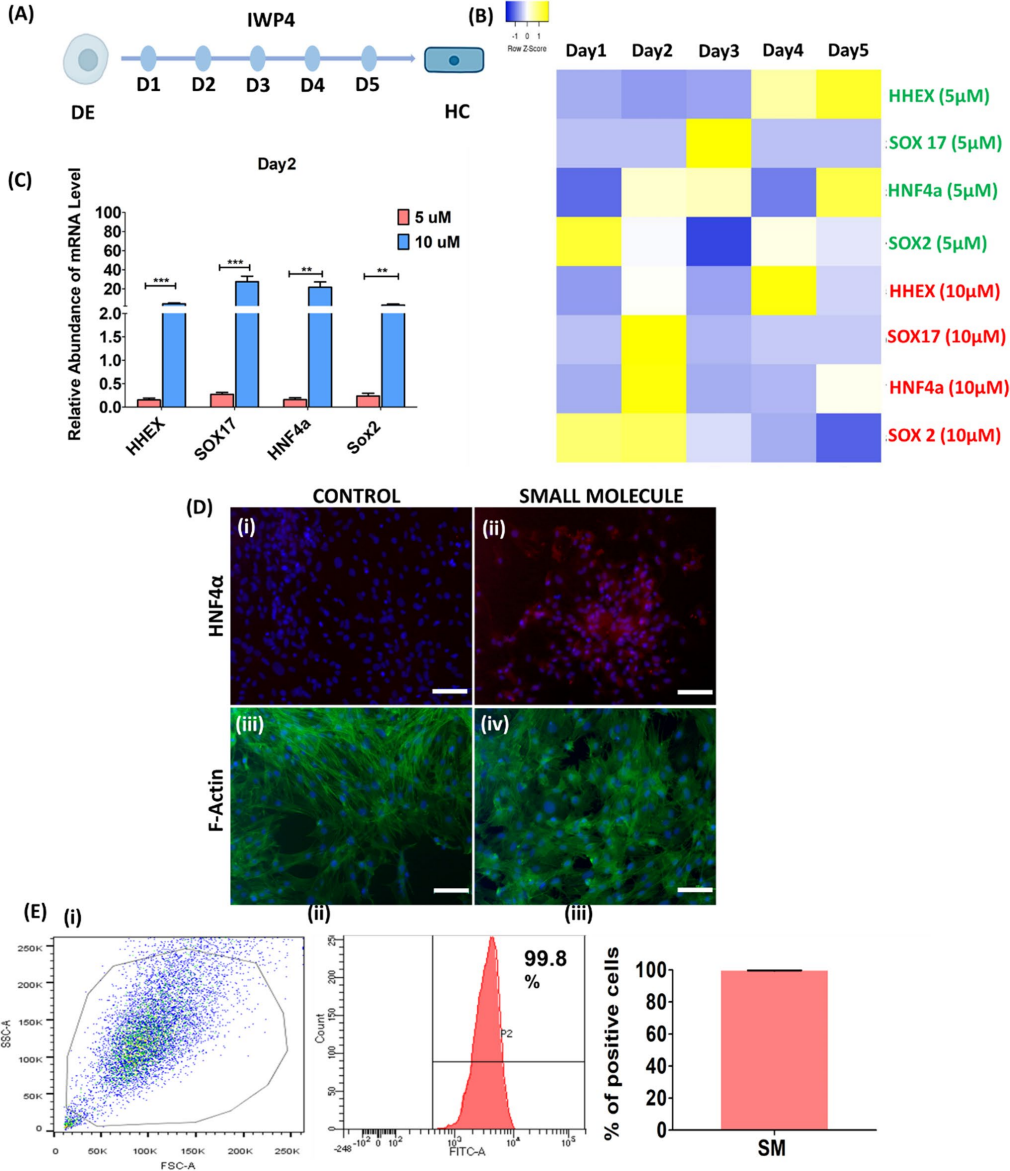
Hepatic Commitment by the Wnt inhibitor IWP4—Stage 2. **A** Schematic representation of hepatic competence (HC) induction in the definite endoderm (DE) using IWP4. DE-primed cells were treated with IPW4 at 5 µM and 10 µM concentrations for 5 days and every 24 h thereafter. HC-specific gene expression was analyzed to optimize the day at which the highest level of HC-specific gene expression was induced. **B** Heatmap analysis of HC-specific gene expression (HHEX, SOX17, HNF4α, SOX2) in IWP4-treated DE cells. IWP4 at a concentration of 10 µM had the highest level of HC-specific gene expression after day 2 of treatment. **C** HC-specific gene expression studied after 2 days of IWP4 (10 µM) treatment showing the highest level of change in the gene expression profile compared to that observed after 5 µM IWP4 treatment. **D** Immunocytochemistry after 2 days of 10 µM IWP4 treatment showing positive staining for HNF4α, confirming the induction of hepatic competent cells. **E** Flow cytometry-based assessment of the efficiency of hepatic competent-specific HNF4α expression (99.8 ± 1%) after 2 days of IWP4 (10 µM) treatment. This finding confirmed the efficient derivation of hepatic competent cells on day 4 of the differentiation protocol, which included 2 days of DE induction and 2 days of HC induction. The data were analyzed by one-way ANOVA. Differences were considered to be statistically significant at *p < .01, **p < .001, and ***p < .0001. The data are presented as the means of three independent experiments; the error bars represent the SDs. Scale bar—100 µM

Upon definitive endoderm induction, specialized cells are formed that commit to the hepatic lineage, eventually forming the liver bud. After approximately E9.5 days of embryonic development until several days, liver progenitors are formed, leading to the formation of the liver. We tested the ability of the Wnt signaling inhibitor IWP4 (5 µM and 10 µM) to induce hepatic competence in the derived DE cells (Fig. 3A). IWP4 induced the expression of genes related to the hepatic competence stage, i.e., HHEX, SOX17, HNF4α and SOX2 (SI Fig. 3). A heatmap analysis of the gene expression profile showed that IWP4 at a concentration of 10 µM had the maximum effect on the hepatic competence-related gene expression of HHEX, SOX17, HNF4α and SOX2 (Fig. 3B) on day 2. HC stage optimization showed that the highest level of gene expression was exhibited by 10 µM IWP4 compared to 5 µM IWP4 on day 2 (Fig. 3C). Immunocytochemistry studies confirmed the protein expression of HNF4α, confirming the hepatic competence achieved by inhibiting Wnt signaling in the DE (Fig. 3D). Compared to those of DE cells, the morphology of hepatic competent cells markedly changed, as assessed by actin filament staining (Fig. 3D (iii), (iv)). This indicated a change in the phenotypic characteristics of the cells and a transition toward a more constricted and epithelial-like phenotype. The differentiation efficiency of the HNF4α protein was 99.8% in DE-treated cells treated with 10 µM IWP4 (Fig. 3E (ii), (iii)). This confirmed the derivation of hepatic competent cells.

### Hepatic Specification by DMSO, β-mercaptoethanol and CHIR 99021: Stage 3

**Fig. 4:**
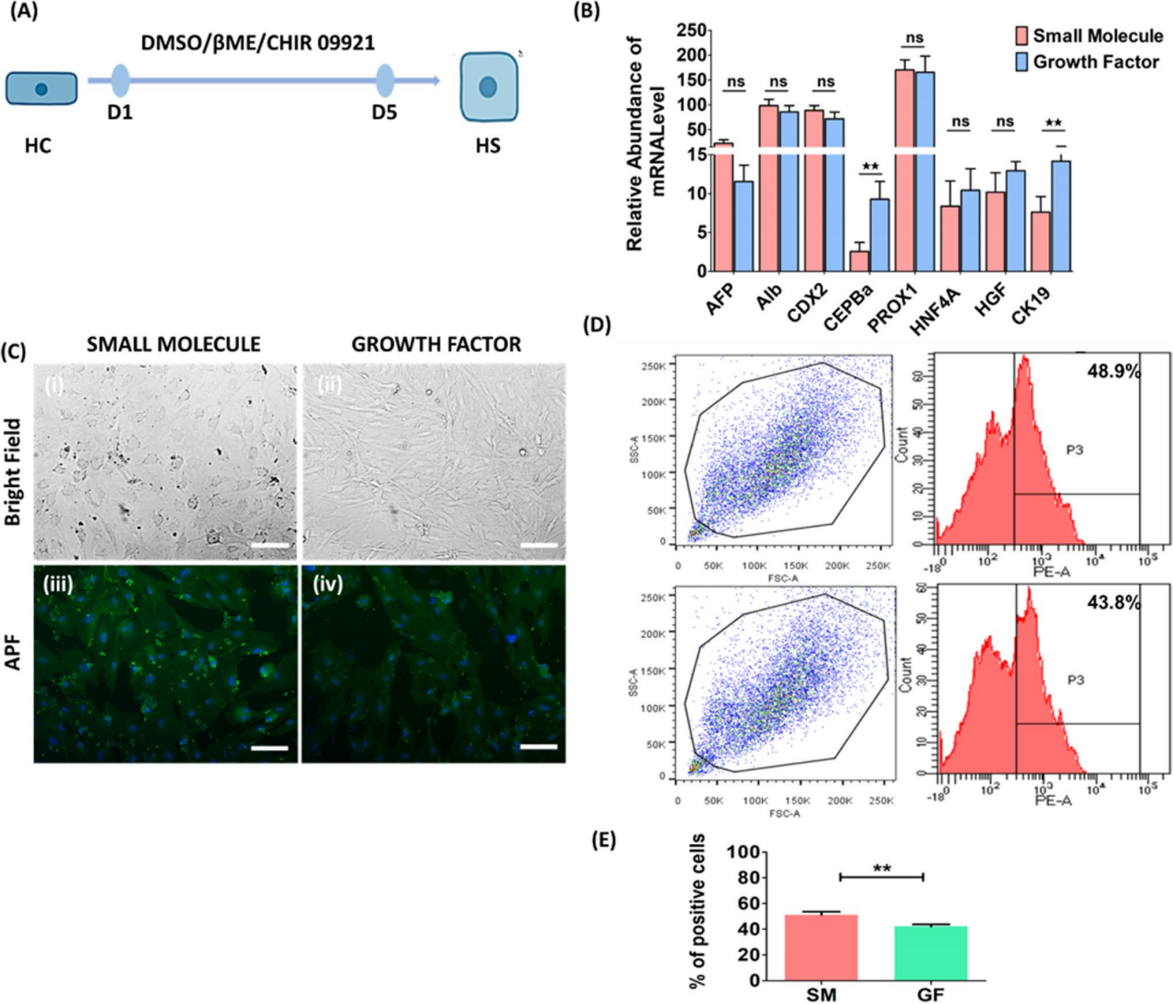
Hepatic specimens treated with DMSO, β-mercaptoethanol and CHIR 99021—Stage 3. **A** Schematic representation of hepatic specification (HS) induction in hepatic competent (HC)-primed cells using DMSO, β-mercaptoethanol, and CHIR 99021 for 5 days. **B** Real-time PCR-based gene expression analysis of the hepatic-specific cells showed that the SM- and GF-treated cells had an HS-specific gene expression profile (AFP, ALB, CDX2, CEPBα, PROX1, HNF4α, HGF and CK19). **C** Immunostaining of HSC markers in SM- and GF-treated cells. bright field image of SM-treated (i) and GF-treated (ii) HS cells. Positive staining of SM-treated HS cells (iii) and GF-treated HS cells (iv). **D** Expression efficiency analysis of HS cells (48.0 ± 2.4%) and GF-treated cells (43.5 ± 3.1%) (ii). Scale bar—100 µM. **E** The bar graph shows that AFP expression was significantly different (*p* < 0.05) from that of the GF protocol. The data were analyzed by one-way ANOVA. Differences were considered to be statistically significant at *p < .01, **p < .001, and ***p < .0001. The data are presented as the means of three independent experiments; the error bars represent the SDs

Specified cells are critical for terminal differentiation of lineage-committed progenitor cells for commitment to hepatocytes. Upon induction of hepatic commitment cells, stage 3 was induced using three chemicals, i.e., dimethylsulfoxide (DMSO), β-mercaptoethanol (βME) and CHIR 99021 (2 µM). DMSO (0.5%), βME (100 nM) and CHIR 99021 (2 µM) were used for 5 days to treat hepatic competent cells derived from stage 2 patients (Fig. 4A). Gene expression analysis revealed induced expression of hepatic-specific genes after 5 days of treatment (Fig. 4B). However, the serum ALB concentration did not significantly differ between SM- and GF-induced patients. CEPBα and CK19 were significantly greater in the GF group (*p* < 0.01) than in the SM differentiation group (Fig. 4B). Immunocytochemical studies of AFP showed positive staining in the SM and GF groups, confirming stage 3-specific protein marker expression. The induction efficiency of stage 3 was studied by analyzing the expression pattern of the stage 3-specific protein AFP. For SM, the AFP expression level was 48.0 ± 2.4% (Fig. 4D (i)); for GF, the AFP expression efficiency was 43.5 ± 3.1% (Fig. 4D (ii)); and there was a significant difference between SM and GF (*p* < 0.05) (Fig. 4E).

### Hepatic Differentiation and Growth—Stage 4

**Fig. 5:**
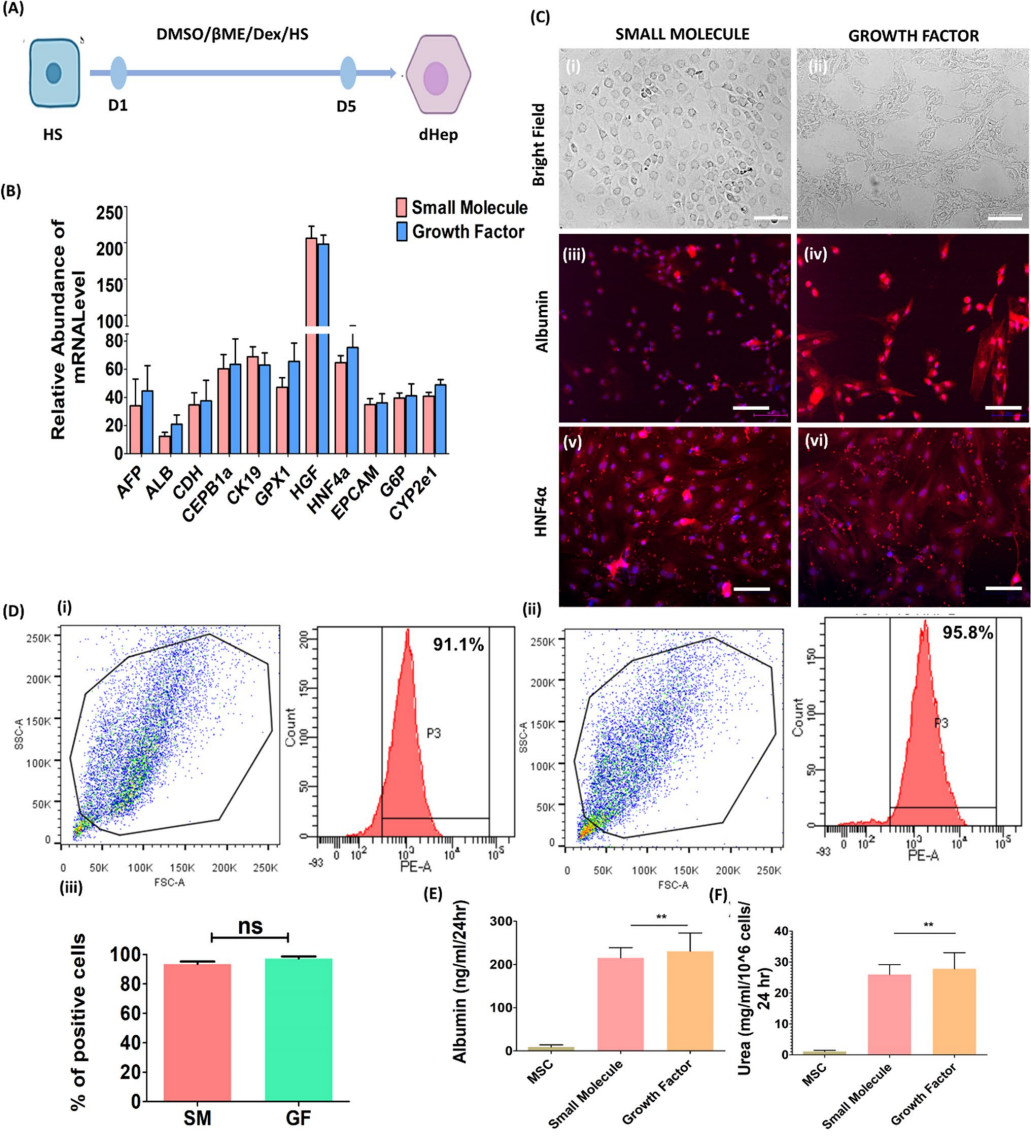
Hepatic differentiation and growth—Stage 4**. A** Schematic representation of hepatic differentiation and growth induction in hepatic specified (HS) cells treated with DMSO, hydrocortisone, dexamethasone and CHIR 09921 for 5 days. **B** Gene expression analysis of the final stage of hepatic differentiation and the growth phase of SM- and GF-treated cells. **C** Microscopic analysis of differentiated hepatocytes after 14 days (for SM) and 28 days (for GF) of culture. Bright-field image of SM-derived dHeps (i) and growth factor (growth factor)- and hepatocyte-like cells (ii). **D** Flow cytometry-based assessment of the expression efficiency of SM in terminally differentiated hepatocyte-like cells revealed 91.1 ± 4% of the total albumin concentration and 95.8 ± 2% of the total albumin concentration according to the GF-based differentiation protocol. Moreover, there was no significant difference between the groups. **E** Functional assessment of differentiated hepatocyte-like cells showing the following characteristics: albumin secretion, MSC-8.99 ± 5.02; SM-215.1 ± 23.61; and GF-230.97 ± 42.21 ng/ml/24 h. and **(F)** urea estimation, MSC -1.02 ± 0.42; SM – 25.95 ± 3.18; GF 27.77 ± 5.19 mg/ml/10^6 cells/24 h. The data were analyzed by one-way ANOVA. Differences were considered to be statistically significant at *p < .01, **p < .001, and ***p < .0001. The data are presented as the means of three independent experiments; the error bars represent the SDs. Scale bar- 100 µm

Expression analyses of terminally differentiated cells were performed to confirm the derivation of dHeps. HS stage-specific cells were cultured to derive differentiated hepatocyte-like cells (dHeps) for 5 days (Fig. 5A). At the end of 14 days, gene expression analysis revealed that the SM-based strategy produced hepatocyte-specific gene expression comparable to that of the GF-based protocol. Interestingly, the expression of the HGF gene was significantly greater in the SM-based protocol than in the GF-based approach (Fig. 5B). SM-derived dHeps exhibited marked morphological changes with loss of the fibroblastic morphology characteristic of MSCs (Fig. 5C (i)). Similar morphological changes were observed in GF-derived hepatocyte-like cells (Fig. 5C (ii)). Immunocytochemistry revealed that, similar to GF-derived hepatocytes, SM-derived dHeps had positive staining for albumin and HNF4α (Fig. 5C (iii, (v)) with a similar trend (Fig. 5C (iv), (vi)). Furthermore, flow cytometry revealed that the expression efficiency of SM-derived dHeps was 91.1 ± 4% that of albumin, while that of the GF-based differentiation protocol was 95.8 ± 2% (Fig. 5D (i), (ii)). Moreover, there was no significant difference in expression efficiency between the SM- and GF-based strategies (Fig. 5D (iii)). Functional assessment of albumin secretion in the supernatant of differentiated hepatocytes revealed that SM was secreted at 215.1 ± 23.61 ng/ml after 24 h. compared to GF-230.97 ± 42.21 and MSC-8.99 ± 5.02. Compared with that of SM, the serum ALB concentration was significantly greater (*p* < 0.001) (Fig. 5E (i)). Similarly, analysis of urea secretion from differentiated hepatocytes revealed that the urea synthesis in SM was 25.95 ± 3.18 mg/ml/10^6 cells/24 h. compared to 27.77 ± 5.19 (GF) and 1.02 ± 0.42 (MSC) mg/ml/10^6 cells/24 h (Fig. 5F (i)). Urea synthesis was significantly greater in GF than in SM and GF (*p* < 0.001).

### Decellularization of the Human Liver

**Fig. 6:**
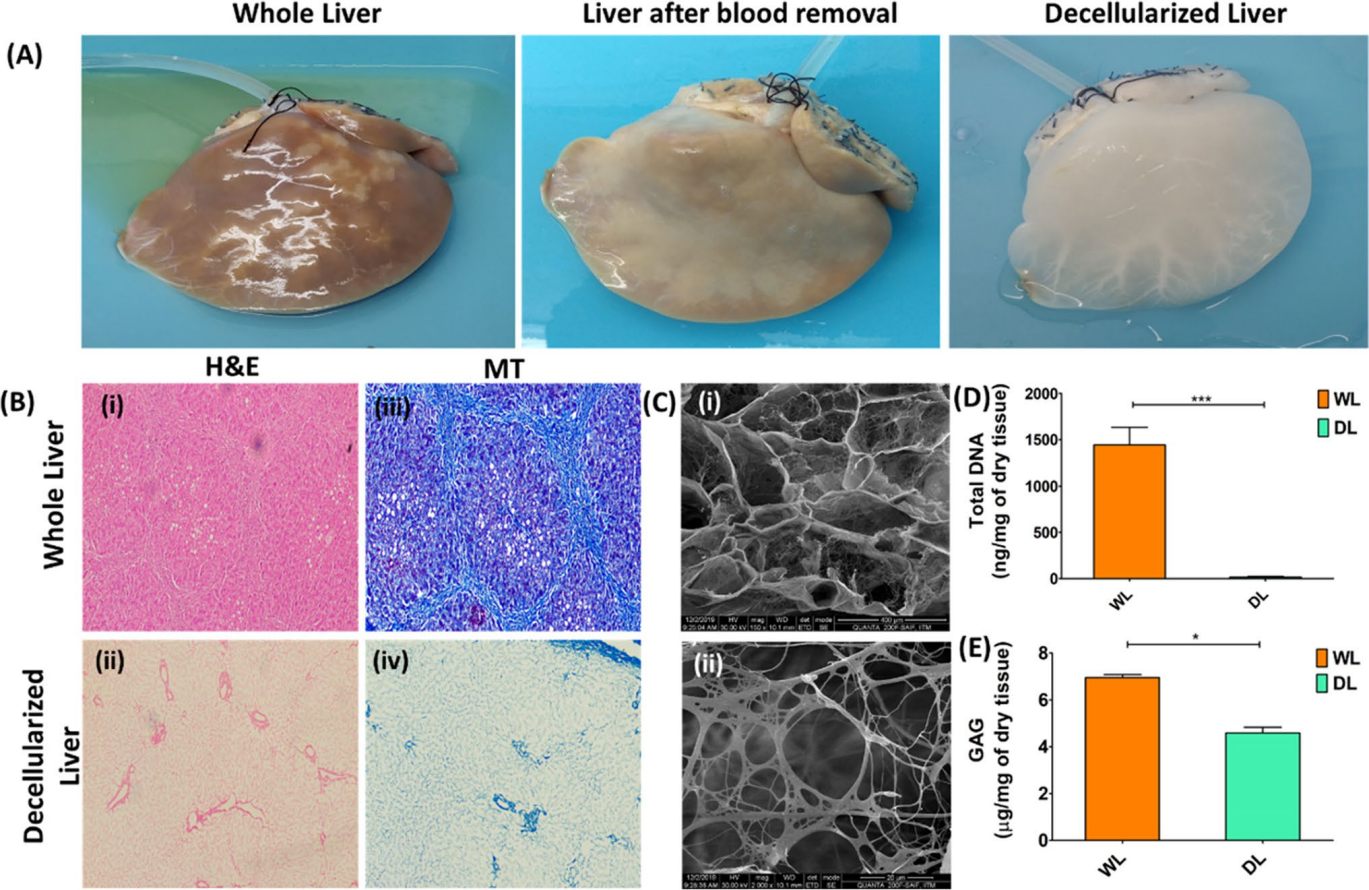
Decellularization of the human liver and characterization of the liver. **A** Cannulated human liver lobe (i), blood removed from the cannulated liver with PBS for 3 h (ii); decellularized liver lobe after 24 h (iii). **B** Histological analysis of the whole liver (WL) and decellularized liver (DL) to confirm successful decellularization of the liver. **C** Scanning electron microscopy (SEM) image of the decellularized liver ECM (DLEM) (i) and magnified image showing the fibrous architecture of the extracellular matrix in the absence of cellular components. **D** The total DNA content of the native liver (1442.0 ± 271.52 ng/mg of dry tissue) was significantly greater (*p* < 0.001) than that of the decellularized liver (17.0 ± 6.36 ng/mg of dry tissue). **E** The total glycosaminoglycan (GAG) concentration estimated from the native whole liver (6.94 ± 0.23 mg/mg of dry tissue) and decellularized liver (DLEM) (4.95 ± .04 mg/mg of dry tissue) showed that the whole-liver GAG concentration (*p* < 0.01) was significantly greater than the DLEM GAG concentration. Scale bars: 400 µm and 20 µm for SEM and 100 µm for H&E and MT staining. The data were compared using the Mann–Whitney U test. Differences were considered to be statistically significant at *p < .05, ***p < .001, and ns–nonsignificant

The gross appearance of the decellularized liver confirmed the removal of cellular materials (Fig. 6A). A histological analysis via hematoxylin and eosin staining confirmed the removal of nuclear and cellular components, as confirmed by the absence of hematoxylin-stained nuclear materials (Fig. 6B (iii)). MT staining revealed the presence of collagen fibers in the decellularized liver matrix while preserving the fibrous collagen structures (Fig. 6B (iv)). SEM imaging revealed the intact fibrous architecture of the decellularized matrix (Fig. 6C (i)) and a magnified image (Fig. 6C (ii)). Total DNA was estimated from the whole liver (1442.0 ± 271.52 ng/mg of dry tissue) and decellularized liver (17.0 ± 6.36 ng/mg of dry tissue), and the results showed that there was a significant difference in total DNA in the DLEM group compared to that in the whole liver (*p* < 0.001) (Fig. 6D). This confirmed that the decellularization technique was successful at removing nuclear materials from the liver. The total GAG content estimated from the whole liver (6.94 ± 0.23 mg/mg of dry tissue) was significantly greater (*p* < 0.01) than that from the decellularized liver (4.95 ± 0.04 mg/mg of dry tissue). This confirmed the loss of GAGs from the decellularized liver compared to that from the native liver as a result of detergent-based perfusion decellularization (Fig. 6E).

### In vitro3D Hepatic Differentiation of MSCs

**Fig. 7:**
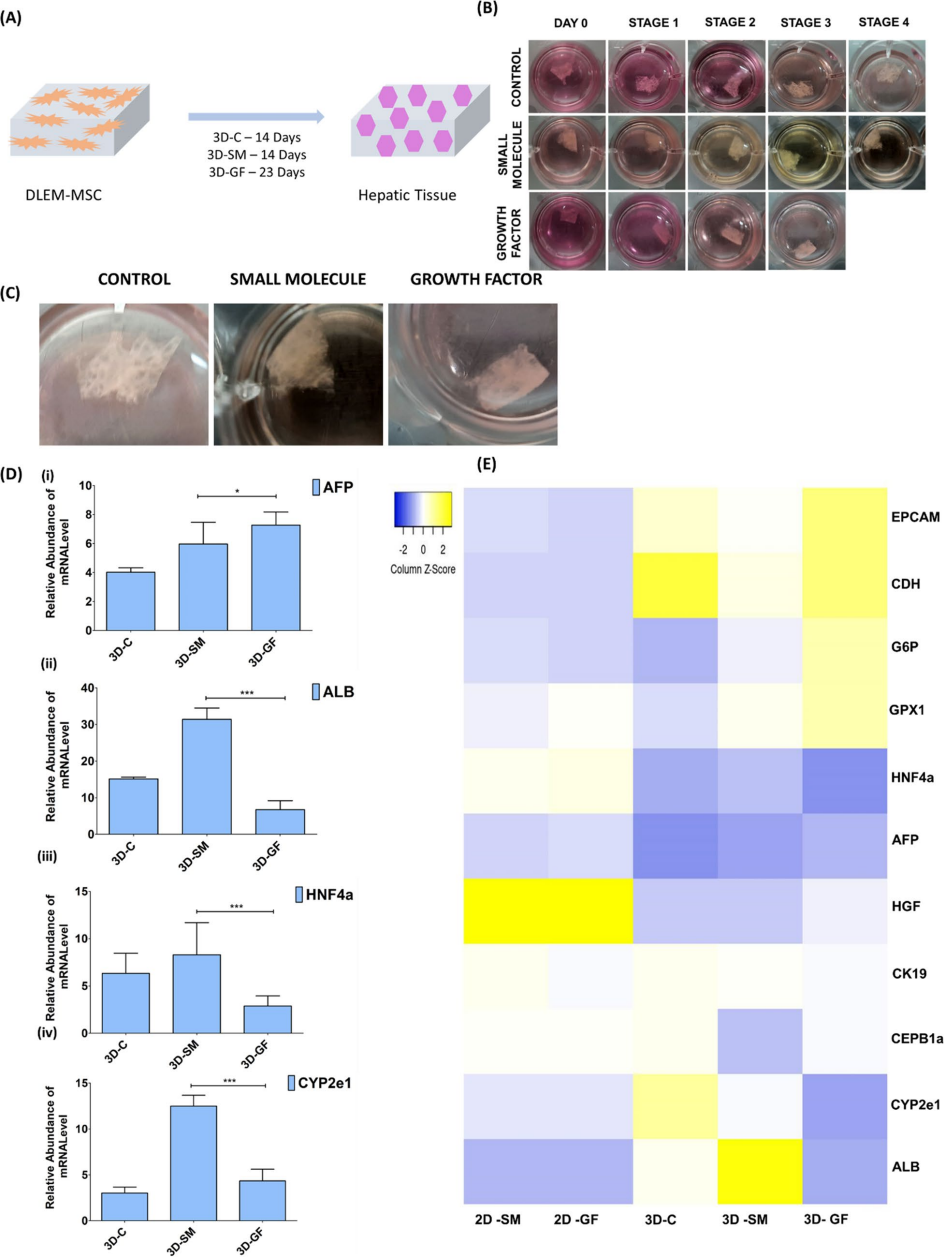
Hepatic tissue development using decellularized liver extracellular matrix (DLEM) and MSCs. **A** Schematic representation of the development of hepatic tissue using SM- and GF-based protocols. **B** Photomicrograph of the different stages of hepatic tissue development obtained using the SM- and GF-based protocols. **C** Photomicrograph of DLEM, 3D-SM and 3D-GF after 14 days and 23 days of differentiation protocol respectively. **D** Real-time analysis of AFP, ALB, HNF4α and CYP2e1 gene expression in control tissue (3D-C), SM-based hepatic tissue (3D-SM) and GF-based hepatic tissue (3D-GF). The AFP level was significantly greater in the 3D-GF group than in the 3D-SM and 3D-C groups. ALB, HNF4α and CYP2e1 were significantly greater in the 3D-SMs than in the 3D-C and 3D-GFs. **E** Heatmap analysis of the hepatocyte-specific genes for SM-derived hepatocytes in 2D (2D-SM), growth factor-derived hepatocytes in 2D (2D-GF), control tissue (MSCs cultured in 2% FBS) without any differentiation factor (3D-C), SM-based hepatic tissue (3D-SM) and growth factor-based hepatic tissue (3D-GF). The data were analyzed by one-way ANOVA. Differences were considered to be statistically significant at *p < .01, **p < .001, and ***p < .0001. The data are presented as the means of three independent experiments; the error bars represent the SDs

MSC-repopulated human DLEM were cultured for 14 days with SM (3D-SM) and growth factor (3D-GF) for 23 days to develop hepatic tissue to study the gene expression profile under 3-dimensional differentiation conditions, and DLEM seeded with MSCs cultured in 2% fetal bovine serum (3D-C) was used as a control (Fig. 7A, B). Figure 7C showed clear photomicrograph of DLEM, 3D-SM and 3D-GF after 14 days and 23 days of hepatic differentiation protocol. Hepatic tissue reconstructed with DLEM and MSCs using an SM-based protocol showed the expression of hepatocyte-specific genes. 3D-GF had higher levels of AFP expression than 3D-SM and 3D-C (Fig. 7D). 3D-SM presented significantly greater levels of gene expression for albumin, HNF4α and CYP2e1 than did 3D-GF and 3D-C (Fig. 7C (ii), (iii), (iv)). Interestingly, 3D-GF presented higher levels of gene expression of genes associated with early stages of liver development, such as CHD, GPX1, and CEPB1α, and genes indicative of progenitors and immature hepatocytes, such as AFP, EpCam, and CK19, than did 3D-SMs (Fig. 7E). The growth factor-based protocol also exhibited a similar gene expression pattern as the SM-based protocol. A heatmap analysis showed enhanced hepatocyte-specific gene expression in hepatic tissue compared to that in differentiated tissue under 2D culture conditions for the SM (2D-SM)- and GF (2D-GF)-based protocols. We also observed hepatocyte-specific gene expression in the MSC-seeded DLEM (3D-C).

### Transplantation of dHep in an ALI Rat Model

**Fig. 8:**
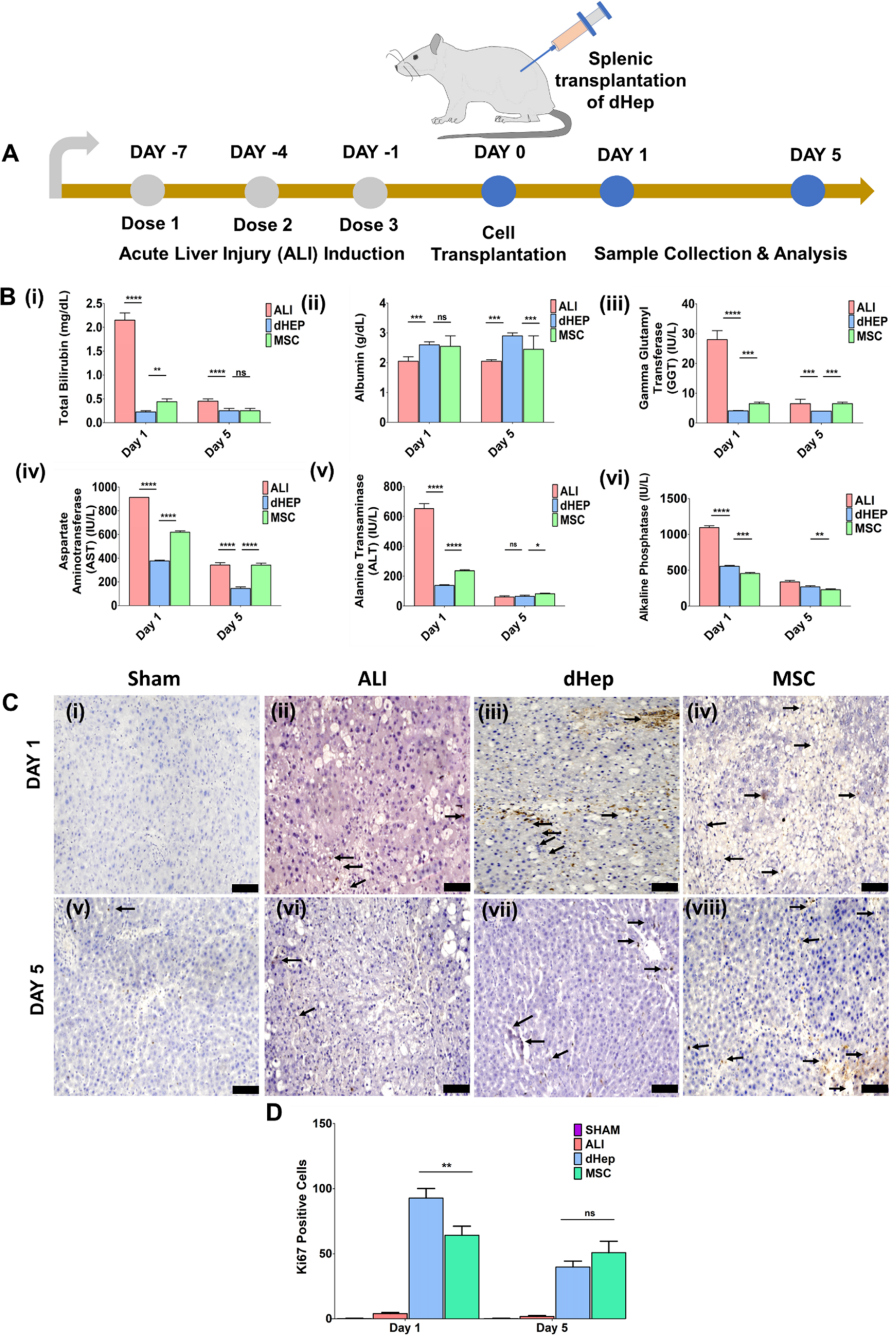
Functional performance of transplanted dHeps in an acute liver injury (ALI) model in rats. **A **Schematic representation of the study design used to assess the functionality of transplanted dHep in a CCl_4_-induced ALI rat model. On Day 0, dHep and MSCs were transplanted into the spleen, and the rats were sacrificed on Day 1 and Day 5 to assess liver function parameters and histological assessment. **B** Evaluation of liver function by quantifying the serum protein concentration and liver-specific chemical profile, such as total bilirubin, albumin, GGT, AST, ALT, and alkaline phosphatase, to assess the therapeutic efficacy of the transplanted dHeps. **C** Evaluation of cell proliferation by Ki67 staining in recipient liver tissue upon dHep and MSC transplantation after day 1 and day 5. Sham control day 1 (i) and day 5 (v); ALI group day 1 (ii) and day 5 (vi); dHep-transplanted group day 1 (iii) and day 5 (vii); MSC-transplanted group day 1 (iv) and day 5 (viii). **D** Percentage of Ki67 positive cells in each group after Day 1 and Day 5. dHep transplanted showed significant difference compared to MSC group, whereas there were no significant differences between dHep and MSC transplanted groups in Day 5. The data were analyzed by one-way ANOVA. Differences were considered to be statistically significant at *p < .01, **p < .001, and ***p < .0001. The data are presented as the means of three independent experiments; the error bars represent the SDs. Scale bar- 800 µm

dHep was transplanted into the spleen of the ALI model mice on day 0 after liver injury induction to assess the therapeutic efficacy of the SM-based treatment. The rats were sacrificed on day 1 and day 5 to assess liver function in all the groups (ALI, dHep and MSC transplanted) (Fig. 8). Total bilirubin (Fig. 8B (i))) and gamma glutamyl transferase (Fig. 8B (ii)) were significantly lower in the dHep-injected group than in the ALI (*p* > 0.001) and MSC (*p* > 0.01) groups after day 1 of dHep transplantation (SI. Table 1). Insulin levels were not significantly different after day 1 of transplantation (Fig. 8B (iii)). There was no significant difference in total bilirubin or direct bilirubin after day 5 of cell transplantation in the ALI, dHep or MSC-transplanted groups (SI Table 1). AST levels were significantly lower in the dHep-transplanted group than in the ALI- and MSC-transplanted groups on day 1 (*p* < 0.001) and day 5 (*p* < 0.001) (Fig. 8B (iv), (v)). ALT was significantly lower in the dHep-transplanted group than in the ALI- and MSC-transplanted groups on day 1 (*p* < 0.001) and day 5 (*p* < 0.01) (Fig. 8B (iv), (v)). There was no significant difference in the serum ALB concentration after day 1 between the dHep- and MSC-transplanted groups, but the difference was significant after day 5 (*p* < 0.01) (Fig. 8B (ii)). To assess the effect of transplanted cells on the regeneration of rat hepatocytes, Ki67 staining was performed and showed significant Ki67-positive staining in the dHep-transplanted groups (Fig. 8C (iii), (vii)) compared to that in the MSC-transplanted group both on day 1 and day 5 (Fig. 8C (iv), (viii)). The ALI model (Fig. 8C (ii), (iv)) also showed less Ki67-positive staining, indicating the proliferation of rat hepatocytes upon liver injury, whereas the sham control showed no positive staining for Ki67 (Fig. 8C (i), (v)) on day 1 or day 5.

**Fig. 9:**
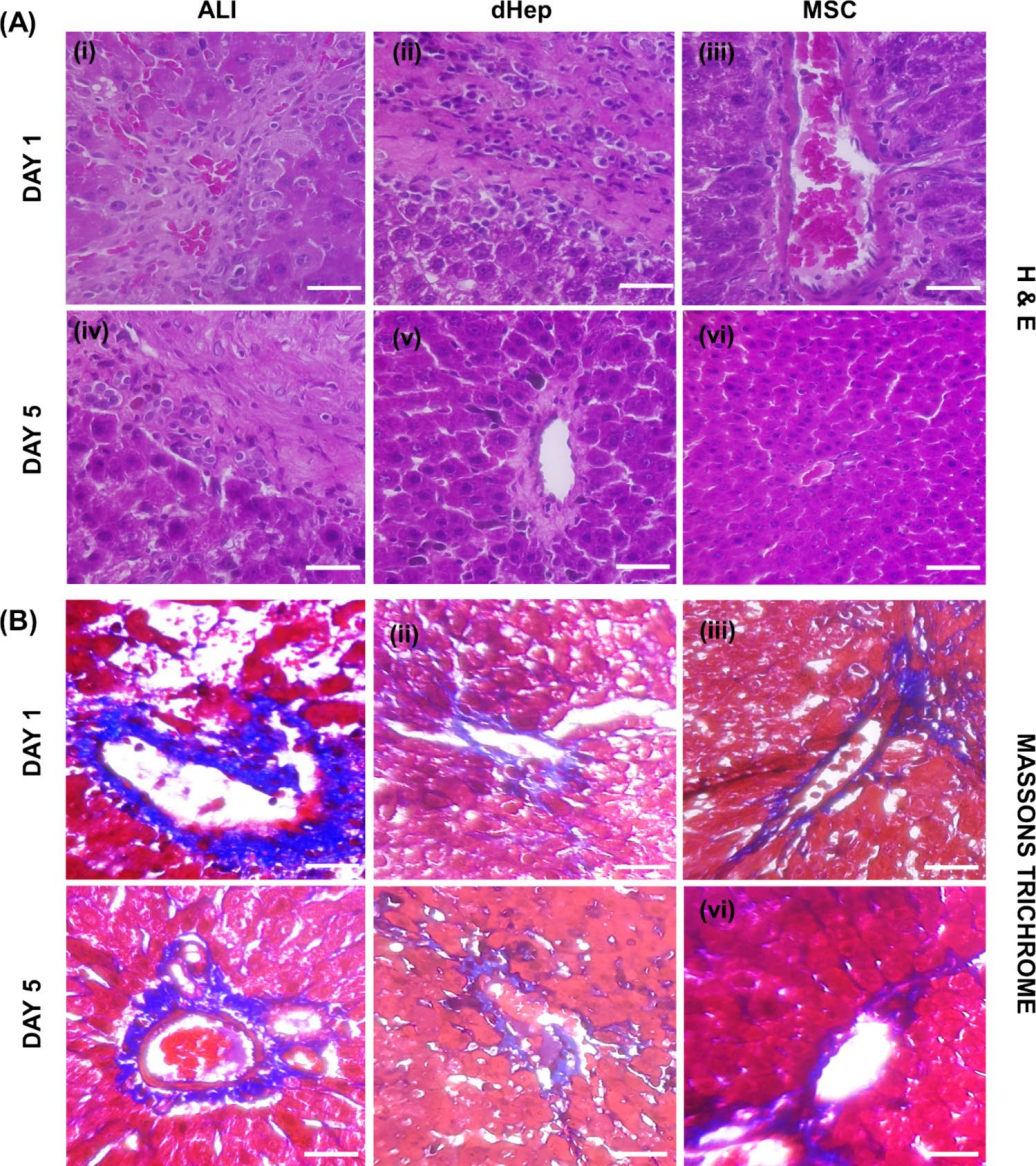
Histological studies of livers retrieved from the ALI, dHep and MSC-injected groups. **A** Hematoxylin and eosin staining of liver tissue from ALI (A (i), (iv)), transplanted dHep (A (ii), (v)) and transplanted MSCs (A (iii), (vi)). On day 1 and day 5, the ALI group exhibited inflammation, portal congestion and sinusoidal congestion. On day 1, the dHep- and MSC-transplanted groups exhibited mild inflammation and portal congestion. Inflammation was resolved after day 5 in the dHep- and MSC-injected groups. **B** Masson Trichrome staining showing the absence of fibrosis in the liver of CCl_4_-treated model rats with ALI. ALI (B (i), (iv)), transplanted dHep (B (ii), (v)) and transplanted MSCs (B (iii), (vi)). Fibrosis was not observed in any of the groups, indicating that the liver injury induction protocol did not have an extensive effect on the liver tissue-induced fibrosis. Scale bar- 320 µm

Histological studies revealed by hematoxylin and eosin staining that the ALI group developed inflammation, portal congestion and sinusoidal congestion on day 1 (Fig. 9A (i), (ii), (iii))) and day 5 post liver injury induction by CCL_4_ (Fig. 9A (iv), (v), (vi)). dHep- and MSC-transplanted groups showed mild inflammation and portal congestion on day 1. Inflammation was resolved after Day 5 in the dHep- and MSC-injected groups (Fig. 9A (v), (vi)). Masson trichrome staining revealed that none of the mice in either the Day 1 (Fig. 9B (i), (ii), (iii)) or Day 5 (Fig. 9B (iv), (v), (vi)) group developed fibrosis as a result of CCL_4_-mediated liver injury.

## Discussion

The development of hepatocyte-like cells is a promising strategy for the treatment of liver failure through the use of cell therapy modules and hepatic tissue development for potential clinical use [1, 31]. However, there are limitations in the differentiation strategy of hepatocytes, such as cells, for the widely used growth factor-based protocol, which is cost intensive and takes anywhere between 23 and 28 days to derive functional hepatocytes, such as cells [9]. Reducing the time needed for deriving hepatocytes like cells and using a strategy that reduces the overall cost of the differentiation process would be highly desirable and have potential clinical applications, as the transplantation of a large number of hepatocytes like cells is required in vivo to manifest therapeutic effects [6, 31]. In this regard, we developed an SM-based differentiation strategy that takes 14 days to derive functional dHeps compared to the growth factor-based protocol, which involves 23–28 days for derivation of functional hepatocytes like cells from MSCs. Therefore, the differentiation process is expensive and time effective.

Hepatocytes arise from the lineage-specific definitive endoderm of the endoderm germ layer. The definitive endoderm gives rise to the liver and pancreas [32]. EGF and bFGF are some of the earliest growth factors known to play critical roles in the developmental pathway of liver organogenesis [33]. The GF-based protocol uses EGF and bFGF to prime stem cells to the definitive endoderm stage [6]. Studies using iPSCs [34], ESCs [35] and MSCs [28, 29, 36] have reported extensive DE derivation via the use of EGF and bFGF. DEs have also been shown to be induced by SM-based approaches, in which Wnt agonists such as CHIR 99021 and BIO have been used [10]. 5 µM or lower CHIR 99021 concentration has been used extensively for definitive endoderm inducing with various stem cells (MSC [37, 38], iPSC [39, 40]). This could be attributed to the fact that during early embryonic development of the liver-specific definitive endoderm, Wnt signaling pathways are highly activated [41]. Therefore, the use of a Wnt agonist seems plausible and has been shown to be sufficient to prime DE-specific cells just by activating Wnt signaling pathways. We used two different concentrations (5 µM and 10 µM CHIR 99021 to optimized DE induction using MSC as the stem cell. We found that 10 µM CHIR 99021 was able to induce definite endoderm-specific gene and protein expression (FOXA2: 99.9 ± 1% SM vs 99.9 ± 1% GF) in bone marrow MSCs. These data are similar to those of other studies in which GFs were used to induce DE in human bone marrow MSCs and iPSCs. This finding is also consistent with the use of a low dose of CHIR 99021 for DE induction in iPSCs [10] and ESCs [40]. However, we observed greater expression of T and SOX17 in the GF group than in the 5 µM CHIR-99021 group on day 2. This difference could be attributed to the differential activation and response of signaling pathways or alternative chromatin states in the SM-treated group, as reported in other publications [42–44]. However, the relative expression of specific genes related to DE may differ among various cell types.

Hepatocytes arise from the endoderm, whereas other nonparenchymal liver cells, such as Kupffer cells and hepatic stellate cells, are sinusoidal endothelial cells that arise from different origins [45]. Therefore, it is essential to study whether the trajectory of differentiation follows a more defined and stage-specific lineage progression associated with hepatocytes and not overlap with that of other nonhepatocyte lineages. The definite endoderm (DE) reflects a more specialized group of cells that give rise to the liver and pancreas. Therefore, the induction of DE is crucial, as supported by the gene and protein expression patterns specific to stage 1 in our study. This was further confirmed by comparison with an extensively established GF-based protocol in which the first stage was DE induction. Furthermore, hepatic competence induction commits DE cells toward hepatic progenitor cells, which further progress toward a hepatic-specific lineage. This stage-specific method of induction overcomes the possibility of cross-lineage cell derivation.

Once DEs are formed, specialized cells commit to gut lineage cells, and these cells progress toward liver formation [32, 46]. At this stage, these cells are known to be competent hepatic cells, and lineage progression is an essential step toward liver organogenesis [33]. In contrast to the GF-based protocol, in which DE cells are directly converted into hepatic-specific cells in stage 2, which is also known as the hepatic specification stage, we first attempted to convert DE-specific cells to a more specialized stage of hepatic competence. This effect was achieved by using IWP4, a Wnt signaling inhibitor. During hepatic competence, the Wnt signaling pathway is tightly and efficiently inhibited, resulting in the hepatic competence of DE cells [47, 48]. IWP4 is a Wnt signaling pathway inhibitor that can be used for hepatic competence induction due to the inhibition of Wnt signaling during the early stage of hepatic competence in the definitive endoderm [49]. We reported the efficient induction of HNF4α by inhibiting the Wnt signaling pathway using IWP4. HNF4α binds to gene clusters with a role in hepatogenesis from the definite endoderm [50]. At the HC stage, HNF4α is highly expressed and governs various aspects, such as the activation of the hepatic-specific gene regulatory network, leading to progression toward a more specialized group of hepatic-specific cells.

Following hepatic competence, lineage-specific cells progress toward the liver toward the specified hepatic cells [32]. In the GF-based protocol, this stage is recapitulated by treating MSCs with HGF and bFGF for 7 days [51]. Alternatively, DMSO has been used efficiently for inducing hepatic specification in iPSC- and ESC-based hepatocyte derivation strategies [10, 52]. DMSO may act as an epigenetic modulator and regulate gene expression levels [35]. Owing to its extensive use in hepatocyte differentiation, it has become a common molecule in the differentiation strategy for hepatocytes. However, there is limited information on its use for deriving MSCs from hepatocytes.

The final stage, which is the hepatic differentiation and growth stage, produces functional hepatocytes, similar to cells. Using a growth factor protocol, this stage was successfully derived based on the early lineage commitment steps recapitulated with gene and protein expression similar to in utero developmental evidence [6]. In our protocol, we used a combination of molecules, including DMSO, dexamethasone, and hydrocortisone, to promote the progression of HS cells toward the final stage of hepatic differentiation and growth (Fig. 5A). In the GF-based protocol, hydrocortisone and dexamethasone are used to promote hepatic differentiation and growth in specified hepatic cells derived at stage 2 of the GF-based protocol. Upon hepatic specification, cells are already committed to the hepatic lineage, and the maturation process is initiated in stage 3 of the GF-based protocol. Similarly, in our strategy, stage 3 involves hepatic specification and is equivalent to stage 2 of the growth factor-based protocol. Therefore, the addition of DMSO during stage 4 of our SM-based strategy may promote efficient induction, as the overall exposure to DMSO, including that in stage 3 and stage 4, increased after 10 days.

In the final stage of differentiation, i.e., hepatic differentiation and growth, we found that the SM-based protocol produced similar hepatocyte-specific gene expression compared to the GF-based protocol. The derived dHeps were functional, as shown by albumin and urea quantitation. Interestingly, there were significant differences in the serum ALB (*p* < 0.001) and urea (*p* < 0.01) concentrations between the SM and GF groups. This difference in functionality could be due to the extended period of differentiation for the GF-based protocol compared to the SM protocol. The final stage is the maturation stage, which requires further investigation and an approach to improve the maturation of functional dHeps.

Differentiation protocols are critical for deriving effective and highly reproducible hepatocytes, similar to cells, for potential clinical applications. However, such protocols behave more efficiently when differentiation is performed under 3-dimensional conditions for the development of hepatic tissue [53]. In this regard, a variety of biomaterial-based approaches have been explored, among which decellularized liver extracellular matrix is one of the most promising biomaterials for accessing native extracellular matrix components of liver tissue biochemically and architecturally [54–56]. In our study, we used human liver-derived decellularized ECM as a natural biomaterial for the development of hepatic tissue and studied the effect of our standardized SM-based protocol on the efficiency of the developed protocol in the development of hepatic tissue. We used ionic (SDS-anionic) and nonionic (Triton X-100) detergent successively to remove the lipids and nuclear materials respectively using perfusion system. SDS has helps in denaturing and removing lipids and lysis of the cells [57, 58] and Triton X100 [59] have been known to remove nuclear materials in decellularization and remnant SDS.

MSC-seeded human DLEMs were cultured for 14 days with SM (3D-SM) for 14 days, and growth factor (3D-GF) was added for 23 days to induce hepatocyte-specific gene expression. Liver-specific ECM promotes hepatogenesis in stem cells possibly by providing a conducive extracellular microenvironment as well as the presence of remnant cytokines and growth factors in the ECM [22, 60]. This was evident in our study, in which 2D-SM and 2D-GF cells exhibited lower gene expression than did 3D-SM and 3D-GF cells (Fig. 7D). Interestingly, we found that 3D-SMs presented significantly greater expression in mature hepatocytes, whereas 3D-GFs presented greater expression of genes involved in the early developmental stages of the liver. This suggested that, compared with the 2D protocol, the GF-based protocol in a 3D environment under static conditions may not efficiently produce differentiated mature hepatocytes. This could be because of factors such as the interaction of GF with liver ECM components and binding to it, which controls the spatiotemporal distribution of GF [11, 61]. In contrast, SMs are stable at 37 degrees and can be pharmacokinetically controlled with minimal interaction with the liver ECM [62, 63].

In vivo transplantation of MSC-derived hepatocyte-like cells has been studied in preclinical models of liver injury, but its therapeutic efficacy in improving liver function loss has failed to be assessed [31, 64]. Our study investigated the therapeutic effect of splenic transplantation of SM-based protocol-derived hepatocytes (dHeps) in an acute liver injury rat model to determine the efficacy of dHeps in alleviating liver injury (Fig. 8A). We used MSCs as an alternative cell modality to compare the therapeutic efficacy of dHep in improving liver injury because they have been extensively used as a cell therapy tool for improving liver regeneration in a liver injury model [65–67]. After day 5, there was no significant difference among the ALI, dHep and MSC groups, suggesting that the liver has its own regenerative potential to ameliorate chemically induced liver injury. The improvement in liver function parameters on day 1 in the dHep-injected group could be attributed to the functionality of dHep, which could have compensated for the acute functional damage induced by CCL_4_. Compared with those in the ALI model, the serum parameters, such as total bilirubin, direct and indirect bilirubin, alanine transaminase (ALT) and aspartate transaminase (AST), in the MSC group significantly decreased compared to those in the ALI model, suggesting regeneration or repair of bone marrow MSCs [68]. However, the serum parameters of the dHep group were significantly lower than those of the MSC group on day 1. This finding suggested the importance of functional hepatocyte requirements for improving liver parameters to ensure rapid therapeutic efficacy. These findings were further supported by the difference in Ki67-positive staining in the liver tissue of dHep recipients compared to that in the MSC-transplanted and ALI models (Fig. 8B). However, both the dHep- and MSC-transplanted groups exhibited significantly greater Ki67-positive staining than did the sham control and ALI groups, suggesting the direct role of the transplanted groups in inducing hepatic regeneration. dHep staining was greater in patches across tissue sections than in those in the MSC groups, suggesting that secretory dHep plays a role in inducing the regenerative capacity of the host liver. There are very few studies in the scientific literature where the differences in the efficacy or superiority of MSC-derived hepatocytes have been compared with those of native MSCs in liver repair and regenerative processes. However, the functional utility of hepatocytes as cells over MSCs could be attributed to the various metabolic and secretory functions of hepatocytes, which are not exhibited by MSCs. MSCs secrete a plethora of factors that induce regeneration in damaged or injured livers, resulting in the growth and proliferation of hepatocytes and thus eliciting an improvement in liver function. However, hepatocytes directly overtake parenchyma function and exhibit improvement in liver function faster than indirect regeneration of the injured liver.

Histological analysis via H&E staining revealed that compared with those in the ALI group, the histological appearance of the dHep- and MSC-injected groups improved (Fig. 9A). This could be due directly to the therapeutic role of dHeps and MSCs. In our study, we found that the HGF gene expression level was significantly greater in these mice than in control mice, which could be one of the reasons for the faster regenerative response. Similarly, MSCs have been shown to act in a paracrine way to manifest their therapeutic effect. Studies have shown that MSCs overexpressing HGF improve liver regeneration in a rat model of liver fibrosis. The authors found that, upon transplantation, HGF-expressing MSCs showed improved serum SGPT compared to that of normal MSCs. The authors inferred that the overexpression of HGF plays a role in the liver regenerative response in a fibrotic rat model [69]. However, the role of HGF secretion from MSC-derived hepatocytes, like cells, has not been studied, and future studies are needed to assess this interesting aspect of its contribution to liver regeneration in vivo. Short-term chemical-induced liver injury does not induce fibrosis [70]. This finding is in line with our observation here that fibrosis was not observed in any of the groups. Fibrosis is one of the hallmarks of chronic liver disease; therefore, SM-derived cells should be studied for use in treating chronic liver disease to reverse or ameliorate the fibrotic response. Our study showed a novel approach for deriving hepatocyte-like cells using SM, which exhibited functionality upon in vivo transplantation in an acute liver injury model in rats.

## Conclusion

For the first time, we used an SM-based approach to derive functional hepatocytes using MSC in a stage-specific manner. This study reduced the overall differentiation time to 14 days compared to 28 days after culture via a growth factor-based protocol. The derived functional hepatocytes showed therapeutic efficacy in an acute liver injury model in rats by improving liver function. We further validated our differentiation strategy by constructing hepatic tissue using human DLEM and found that an SM-based approach resulted in hepatocyte-specific gene expression similar to that of a growth factor-based protocol in 14 days. Our work has potential clinical application, as MSCs are currently being explored extensively in clinical application as a stem cell source owing to its genomic stability compared to pluripotent stem cells. Further, the reduced time needed for hepatocyte differentiation using SM decreases the cost and improves time effectiveness for immediate application.

## Supplementary Information

Below is the link to the electronic supplementary material.

ESM 1 (DOCX 614 KB)

## Data Availability

The data that support the findings of this study are available in the manuscript.
