# Supplementary material for: Small Molecule-Mediated Stage-Specific Reprogramming of MSCs to Hepatocyte-Like Cells and Hepatic Tissue for Liver Injury Treatment
Source: Stem Cell Rev Rep. 2024 Sep 11;20(8):2215–35. doi: 10.1007/s12015-024-10771-x (PMC11554881; doi:10.1007/s12015-024-10771-x)
Supplement: Supplementary file 1 — (DOCX 614 KB) [file 12015_2024_10771_MOESM1_ESM.docx]

**Supplemental Information (SI)**

**Small Molecule Mediated Stage Specific Reprogramming of MSC to Hepatocytes Like Cells and Hepatic Tissue for Liver Injury Treatment**

Santosh Gupta^1,2#^, Akriti Sharma^1^, Muthukumarassamy Rajakannu^3^, Jovana Bisevac^2^, Mohamed Rela^3^, Rama Shanker Verma^1#^

^1^Stem Cell and Molecular Biology, Laboratory, Department of Biotechnology, Bhupat and Jyoti Mehta School of Biosciences. Indian Institute of Technology Madras, Chennai, Tamil Nadu, India 600036

^2^Centre for Eye Research and Innovative Diagnostics, Department of Ophthalmology, Institute of Clinical Medicine, University of Oslo, Oslo, Norway

^3^The Institute of Liver Disease & Transplantation, Dr. Rela Institute & Medical Centre, Bharath Institute of Higher Education & Research, Chromepet, Tamil Nadu, India

1. **Methods**

**1.1 Rat Bone Marrow Mesenchymal Stem Cells isolation and characterization**

Wistar rats were purchased from an authorized breeder (Animal Breeder Name, Chennai, TN, India). The animal study was approved by the Institute Animal Ethical Committee (IAEC), Indian Institute of Technology Madras, India. The rats were sacrificed by ether overdose and the body was sterilized thoroughly using 70% Isopropanol solution. The hind limb was clipped and skin was peeled off to muscles were removed to expose the bone. The bone segment was removed from the pelvic joint and the segregated bone (tibia and femur) was isolated in a sterile DPSA buffer. The epiphysis of the long bones was cleared and cut off using a scalpel blade. The medulla was flushed with 3ml of DMEM-F12 media containing 10% FBS and 1%Pen-Step solution using a 18G gauge needle syringe. Medium and cells were gently pulled up and down many times on ice using the same needle and syringe to form a single-cell suspension. The bone marrow suspensions were grown on polystyrene six-well dishes, and non-adherent cells were eliminated after two days using a series of PBS washes and subsequent medium changes. Adherent cells were grown in monolayer cultures in 5% CO2/95% air atmosphere at 37°C with complete media replacement done every 3 days. The first population of cells were labelled as P0. The confluent cells were dissociated with 0.25 % trypsin and 0.01 % EDTA and subcultured at a density of 5x10^4^ cells per well in new six-well culture dishes. The cultures were referred to as P1, P2, P3, P4, and P5 after they were replicated four times and used for downstream studies for differentiation and in vivo work. Cells were split upon achieving 80% confluency.

**Differentiation studies**

All the differentiation studies were performed using cells up to P6.

**1.2 RNA isolation and Semi quantitative Real Time-PCR**

Total RNA was isolated from cells using TRIzol according to the manufacturer’s protocol and quantified using nanodrop (spectrophotometer). cDNA was converted using 2µg of total RNA using MMLV-RT enzyme (Thermo Fisher Scientific, USA) and oligo-dT primers (New England Biolabs, USA). Real Time PCR was performed using SYBR Green in an ABS RT semi quantitative system (USA). SYBR green assay were used to assess the gene expression using GAPDH as an endogenous control (See table no – 5) for the list of primers and its sequence used in this study). Expression level were quantified relative to GAPDH and normalized to undifferentiated BM MSC controls sample cultured in 2% FBS in alpha MEM media supplemented with 100mM Glutamine, 1X Nonessential amino acids (NEAA), 1X Sodium Pyruvate, and 1x Penicillin-Streptomycin. Results were shown as the mean of three independent experiments. Error bar represents standard deviation.

**1.3 Heat Map Analysis and Data Generation**

Heat map was generated by using HeatMapper with semi quantitative RT PCR data for all the groups in stage 1, stage 2 and hepatic tissue. Data were used in triplicate to generate heat map.

1. **Results**


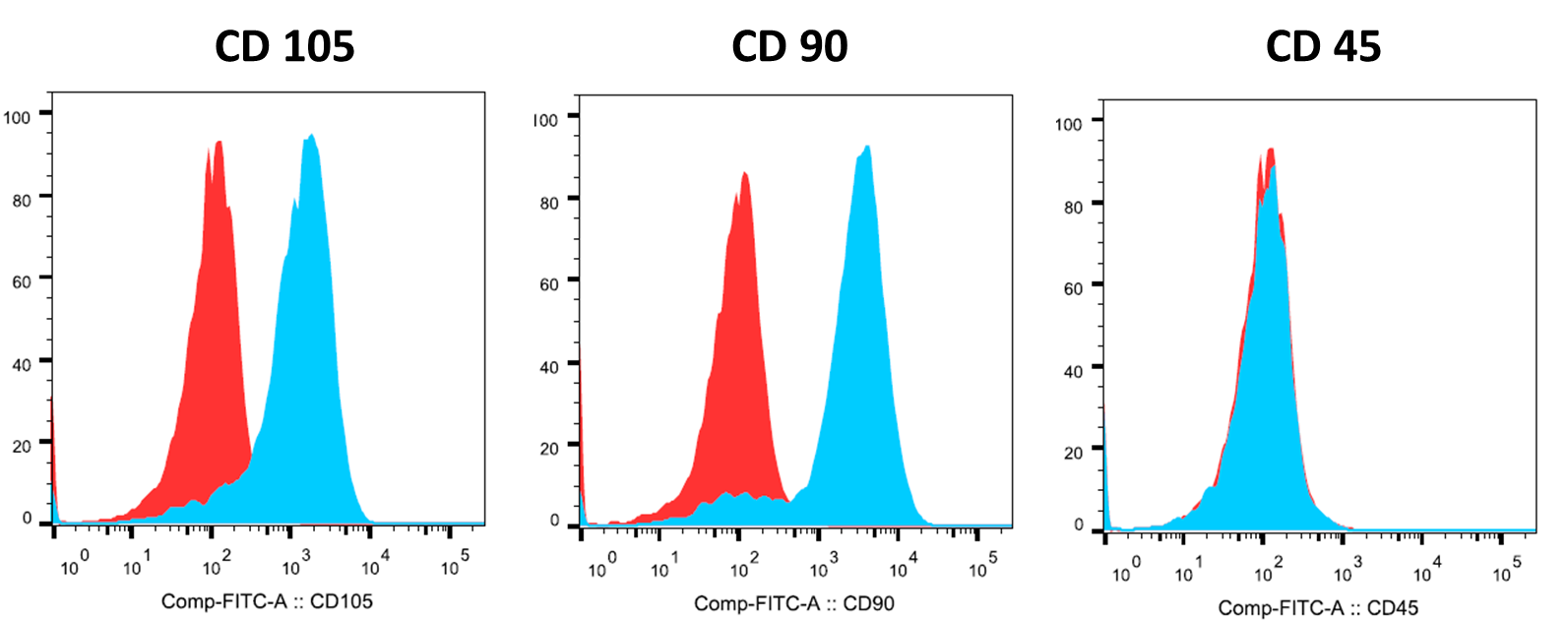


**SI Figure 1** – Characterization of rat BMSC for its multipotency and expression pattern based on ISCT guidelines. (A) Phenotypic surface expression of rat BMSC indicating 78.7% of CD 105+, 86.9% CD 90+ and 0.88% + for CD 45+ respectively. Red colour curve represents unstained rat BMSC and blue colour curve represents rat BMSC stained for different surface markers (Positive marker - CD 105, CD 90; Negative marker – CD 45).


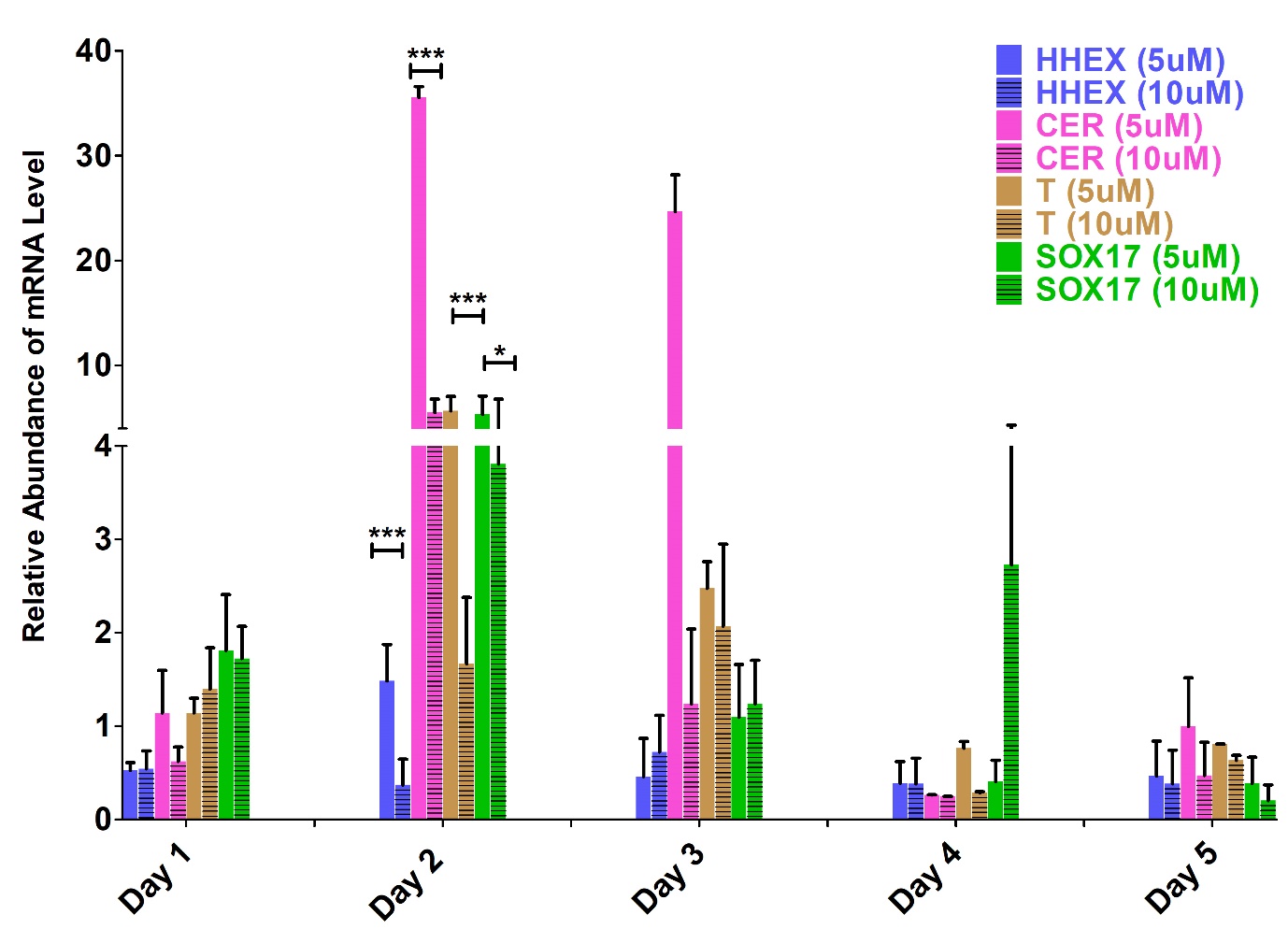


**SI Figure 2** – Definitive endoderm (DE) Induction by CHIR 99021 – Stage 1. DE specific gene expression analysis from day 1 to day 5 of 5µM, 10 µM CHIR 99021. Day 2 gene expression was significantly higher compared to Day1, Day3, Day4 and Day5. Between 5µM and 10 µM CHIR 99021, 5µM treatment induced significantly higher level of HHEX, CER, T and SOX17 genes than 10 µM CHIR 99021. Thus, Day 2 with 5µM CHIR 99021 induction was optimized for derivation of definitive endoderm. The gene expression was normalized with MSC as the control. GAPDH was used was endogenous control.


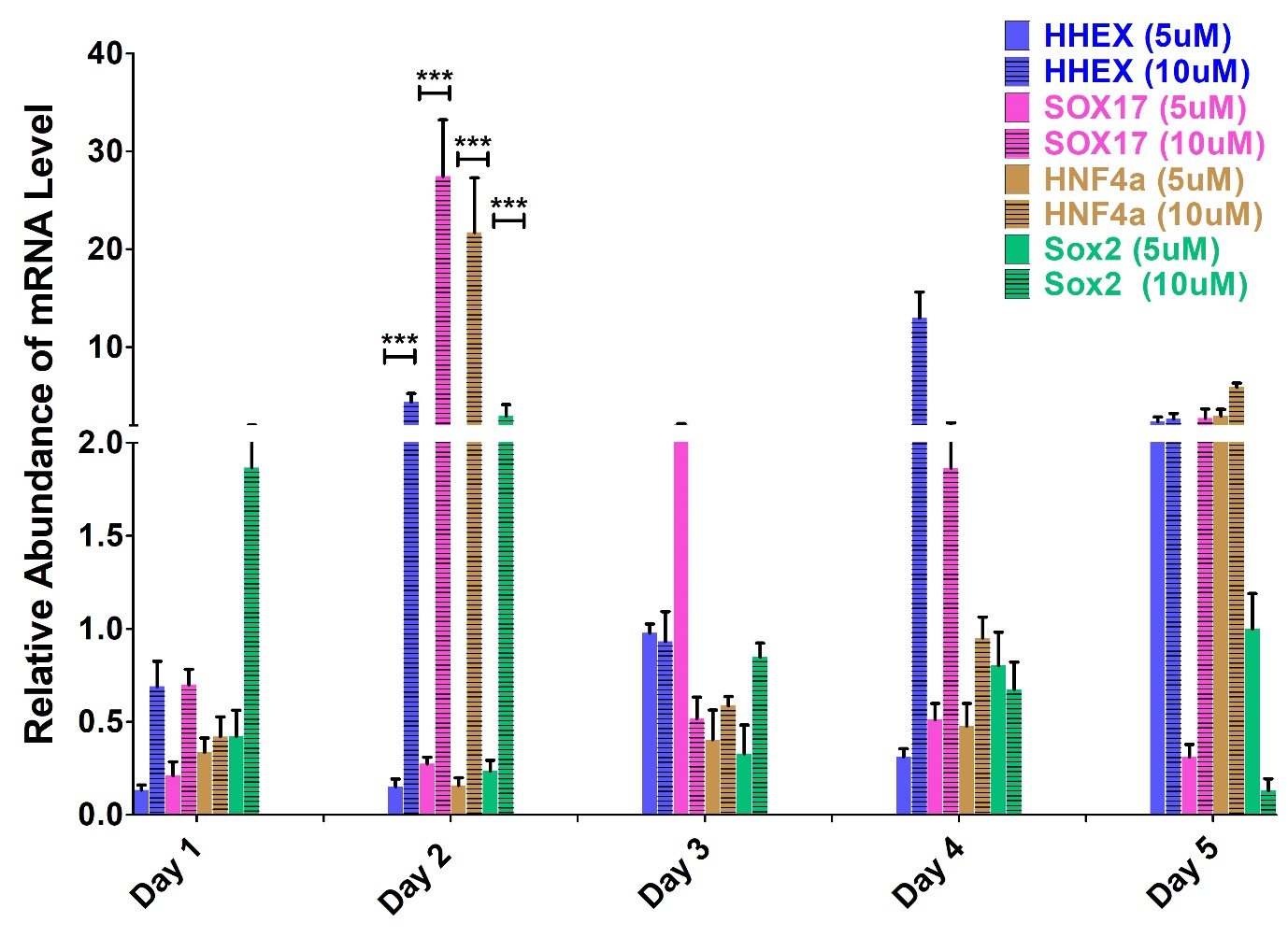


**SI Figure 3 -** Hepatic Commitment (HC) by wnt inhibitor IWP4 - Stage 2. (C) HC specific gene expression studied from Day 1 to Day 5 of 5µM, 10 µM IWP4. Day 2 gene expression was significantly higher compared to Day1, Day3, Day4 and Day5. Between 5µM and 10 µM IWP4, 10µM treatment induced significantly higher level of HHEX, SOX15, HNF4a and SOX2 genes than 5 µM IWP4. Thus, Day 2 with 10µM IWP4 induction was optimized for derivation of definitive endoderm. The gene expression was normalized with MSC as the control. GAPDH was used was endogenous control.

**SI Table 1** - Serum parameters assessed for studying the therapeutic effect of transplanted dHep in an Acute liver Injury (ALI) model of rat.

| Serum Parameters | ALI (Day1) | ALI (Day5) | dHEP (Day 1) | dHEP (Day 5) | MSC (Day 1) | MSC (Day 5) |
| --- | --- | --- | --- | --- | --- | --- |
| Bilirubin Total | 2.15 ± 0.21 | 0.45± 0.07 | 0.225± 0.03 | 0.25± 0.07 | 0.44± 0.08 | 0.25± 0.07 |
| Bilirubin Direct | 1.38± 0.11 | 0.12± 0.02 | 0.11± 0.01 | 0.125± 0.03 | 0.29± 0.15 | 0.15± 0.07 |
| Bilurubin Indirect | 0.77± 0.09 | 0.33± 0.04 | 0.095± 0.01 | 0.125± 0.03 | 0.15± 0.07 | 0.15± 0.07 |
| Alkaline Phosphatase (ALP) | 1094.5± 36.0 | 337.5± 28.9 | 555.5± 16.2 | 269± 21.2 | 454.5± 19.0 | 228± 18.3 |
| Aspartate Aminotransferase (AAT) | 913± 0 | 342± 28.2 | 377± 9.89 | 145± 18.3 | 619± 16.9 | 341.5± 23.3 |
| Alanine Transaminase (ALT) | 652± 46.6 | 60.5± 10.6 | 137.5± 6.36 | 65± 9.8 | 235.5± 9.1 | 82± 5.6 |
| Gamma Glutamyl Transferase (GGT) | 28± 4.2 | 6.5± 2.1 | 4.1± 0.14 | 4± 0 | 6.5± 0.7 | 6.5± 0.7 |
| Protein Total | 6.1± 0.28 | 7.6± 0.28 | 7.45± 0.07 | 7.25± 0.21 | 7.35± 0.07 | 7.6± 0.28 |
| Albumin | 2.05± 0.21 | 2.05± 0.07 | 2.6± 0.14 | 2.9± 0.14 | 2.55± 0.49 | 2.45± 0.63 |
| Globulin | 4.05± 0.49 | 5.55± 0.35 | 4.85± 0.07 | 4.35± 0.07 | 4.8± 0.56 | 5.15± 0.91 |
| Albumin / Globulin Ratio | 0.51± 0.11 | 0.37± 0.04 | 0.54± 0.02 | 0.665± 0.02 | 0.54± 0.16 | 0.49± 0.21 |

ALI – Acute Liver Injury; dHep – Small molecule differentiated Hepatocytes like cells; MSC – rat bone marrow derived mesenchymal stem cells.

**SI Table No. 2 *- Stage Specific gene analysis for semi quantitative RT PCR and HeatMapper data analysis***

| **STAGE 1** | **STAGE 2** | **STAGE 3** | **STAGE 4** |
| --- | --- | --- | --- |
| CER1  HHEX  SOX17  T(TBXT) | SOX17  SOX2  HHEX  HNF4α | AFP  ALB  PROX1  CEBPA  HGF  CDX2  HNF4α  CK19 | ALB  AFP  TBX3  CEBPA  CK19  CDH  HNF4α  GPX1  CYP2e1  HGF  EPCAM |

**SI Table No. 3- *Stage Specific Protein analysis for Immunocytochemistry***

| **STAGE 1** | **STAGE 2** | **STAGE 3** | **STAGE 4** |
| --- | --- | --- | --- |
| FOXA2 | HNF4a | AFP | ALB |

**SI Table No. 4-  *Stage Specific Protein analysis for Flow Cytometer study***

| **STAGE 1** | **STAGE 2** | **STAGE 3** | **STAGE 4** |
| --- | --- | --- | --- |
| FOXA2 | HNF4a | AFP | ALB |

**SI Table No. –5 - *Chemicals used for differentiation***

| **Name** | **Sources** | **Purity** | **Solvent** | **Stages of Protocol used** | **Concentration Used** |
| --- | --- | --- | --- | --- | --- |
| CHIR99021 | Sigma-Aldrich | >95% | DMSO | Stage I | 5µM and 10 µM |
| IWP4 | Tocris | >95% | DMSO | Stage II | 5µM and 10 µM |
| C59 |  | >95% | DMSO | Stage II | 1 µM, 5µM, 10 µM |
| B27 |  |  |  | Stage I & II | 1X |
| DMSO | Merck | >99% | - | Stage III | 0.5% |
| β-Mercaptoethanol |  | >99% | - | Stage III | 100 nM |
| Dexamethasone | Sigma-Aldrich | >95% | Ethanol | Stage IV | 100 nM |
| Hydrocortisone | Sigma-Aldrich | >95% | DMSO | Stage IV | 100 nM |
| Insulin-Transferrin-Selenium (ITS) | Gibco |  |  |  | 1X |
| Knockout Serum (KOSR) | Gibco |  |  |  | 20 % - Stage III  1% - Stage IV |

**SI Table No. 6 *- List of Primers***

| **Target** | **Froward Sequence (5’-3’)** | **Reverse Sequence (5’-3’)** |
| --- | --- | --- |
| FOXA2 | GGTGGGTAGCCAGAAAAAGGC | CGTGCCCTTCCATCTTCACG |
| CER1 | CCAAATTCACCACCACGCAC | CGACGCTCCTCTCCATGTTC |
| GATA4 | GCCTAGAGGCTGTTCTGTCC | GGCTAGAGGTGCCTAGTCCT |
| HHEX | ACGTTTTCCGTACCGGTGTT | ATGCTGGCCCCCTTTCTAAG |
| T (TBXT) | TCTAGGCACAACATGGCAGG | AAATTGGACCACAGCCTCGT |
| SOX17 | TGGTCCACTCACAACTGCTG | GGGGAAATAGGACGGCTGAA |
| SOX2 | ACAGCATGTCCTACTCGCAG | ATGCTGATCATGTCCCGGAG |
| HNF4 | TGACTCTCGGGGTCGTTTTG | CCCCAGAGTTACAGACCTCCA |
| AFP | ACCATCGAGCTCGGCTATTG | CGTTTCAGACTGGGAGCACT |
| ALB | CGTGGACAAGTGTTGCAAGG | GTGGCCTGAGATGGTTGTGA |
| PROX1 | TTGACTCGGGACACAACGAG | TGATAGCCCTTCATTGCGCT |
| CEBPA | CCATCCGCCTTGTGTGTACT | TAGACGCGCACACTGACATT |
| CDX2 | GAGCCAAGATGGCTGTTTCC | AGGTTCTGCAGACTTCGGTC |
| EPCAM | ATTGCGGGGATTGTTGTCCT | ATCCTCCCCAGGTCTATCCG |
| CK19 | GAGATCGCCACCTACCGAAG | GGAAGGGCTGGTGTGAACTT |
| G6P | AATGAACGTGCTCCACGACT | CTGCCACCCAGAGGAGATTG |
| HGF | ACAGCTTTTTGCCTTCGAGC | GCAGGTCATGCATTCAACTTCT |
| CYP2e1 | TAATGGGCCCACATGGAAGG | GCGCAGCCAATCAGAAATGT |
| GAPDH | AGTGCCAGCCTCGTCTCATA | GATGGTGATGGGTTTCCCGT |

**SI Table No. – 7 *- List of antibodies***

| **Sr. No.** | **Protein** | **Clone** | **Dilution** |
| --- | --- | --- | --- |
| 1 | Forkhead Box A2 (FOXA2)-anti-rabbit (Millipore, USA) | 9HCLC | 1:500 |
| 2 | Hepatocytes Nuclear Factor (HNF) 4a anti-rabbit (Thermo Scientific, USA) | SN72-03 | 1:500 |
| 3 | Albumin-anti-mouse (Millipore, USA) | Polyclonal | 1:800 |
| 4 | Alpha Fetoprotein (AFP) (Millipore, USA) | 6E6 | 1:500 |
| 5 | Secondary Anti Rabbit 555 (Thermo Scientific, USA) | Polyclonal | 1:1000 |
| 6 | Secondary Anti Rabbit 488 (Thermo Scientific, USA) | Polyclonal | 1:1000 |
| 8 | Secondary Anti Mouse 488 (Thermo Scientific, USA) | Polyclonal | 1:1000 |
| 9 | Secondary Anti Mouse 555 (Thermo Scientific, USA) | Polyclonal | 1:1000 |
